# In Situ Transformable Nanoparticle Effectively Suppresses Bladder Cancer by Damaging Mitochondria and Blocking Mitochondrial Autophagy Flux

**DOI:** 10.1002/advs.202409425

**Published:** 2024-12-09

**Authors:** Yulin Lv, Benli Song, Guang Yang, Yuting Wang, Zeyu Wu, Minggui Si, Zongzheng Yang, Huilin Chen, Chen Liu, Min Li, Yinshi Zhang, Zengying Qiao, Lu Wang, Wanhai Xu

**Affiliations:** ^1^ NHC Key Laboratory of Molecular Probes and Targeted Theranostics Harbin Medical University Harbin 150001 China; ^2^ Department of Urology The 2nd Affiliated Hospital of Harbin Medical University Harbin 150001 China; ^3^ Department of Urology Harbin Medical University Cancer Hospital Harbin 150001 China; ^4^ Key Laboratory of the Fourth Hospital of Harbin Medical University Harbin 150001 China; ^5^ CAS Center for Excellence in Nanoscience CAS Key Laboratory for Biomedical Effects of Nanomaterials and Nanosafety National Center for Nanoscience and Technology (NCNST) No. 11 Beiyitiao Zhongguancun Beijing 100190 China; ^6^ Department of Neurosurger The First Affiliated Hospital of Harbin Medical University Harbin 150001 China

**Keywords:** lysosomal escape, mitochondria damage, mitochondrial autophagy, self‐assembly, sonodynamic therapy

## Abstract

Tumor therapeutic strategies based on mitochondrial damage have become an emerging trend. However, the low drug delivery efficiency caused by lysosomal sequestration and the activation of protective mitochondrial autophagy severely restricts the therapeutic efficacy. Herein, an in situ transformable nanoparticle named **KCKT** is developed to promote lysosomal escape and directly damage mitochondria while blocking mitochondrial autophagy. **KCKT** exhibits acid responsiveness for precise self‐assembly into nanofibers within the lysosomes of cancer cells. The massive accumulation of nanofibers and excessive production of reactive oxygen species (ROS) under sonodynamic therapy synergistically induce lysosomal damage. This facilitates the escape of nanofibers from lysosomal sequestration, thereby enhancing drug delivery. Subsequently, the escaped nanofibers specifically aggregate around the mitochondria for long‐term retention and generate ROS under ultrasound irradiation to induce mitochondrial damage. Notably, due to lysosomal dysfunction, damaged mitochondria cannot be cleared by autophagy, further aggravating oxidative damage. These results reveal that **KCKT** effectively improves drug delivery and mitochondria‐targeted therapy efficiency by blocking protective autophagy. These findings hold significant potential for advancing the field of mitochondria‐targeted therapy.

## Graphical abstract

An in situ transformable nanoparticle with pH responsive and sonodynamic therapy capabilities is developed for the treatment of bladder cancer. Under ultrasound irradiation, the nanoparticle effectively facilitates lysosomal escape, disrupts mitochondrial function, and blocks mitochondrial autophagy flux, which demonstrates significant potential for improving drug delivery and the tumor therapeutic effect based on mitochondrial damage.

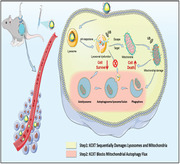

## 1 Introduction

Mitochondria serve as the energy factories of cells, regulating cellular environmental homeostasis and energy metabolism.^[^ ^1^ ^]^ The impairment of mitochondrial activity in malignancies could inhibit tumor proliferation, migration, and drug resistance.^[^ ^2^ ^]^ Therapeutic strategies based on mitochondrial damage have become the focus.^[^ ^3^ ^]^ Several clinical trials are investigating the potential of inhibiting mitochondrial function as a novel tumor therapy.^[^ ^4^ ^]^ However, tumors have evolved mechanisms to adapt to the challenging conditions of their surroundings. For example, when mitochondrial damage occurs, tumor cells spontaneously activate mitochondrial autophagy in response to cellular injury.^[^ ^5^ ^]^ During this process, damaged mitochondria are selectively enveloped by autophagosomes and then merged with lysosomes. This fusion event facilitates the comprehensive degradation of the mitochondria, ensuring the preservation of cellular environmental homeostasis.^[^ ^6^ ^]^ Consequently, protective mitochondrial autophagy can significantly reduce the therapeutic effectiveness of mitochondrial damage.^[^ ^7^ ^]^ Furthermore, although the combined therapy of autophagy inhibitors and antitumor drugs has shown promising results, the disadvantages of autophagy inhibitors, such as poor tumor distribution, limited cellular permeability, and serious side effects, limit their further application.^[^ ^8^ ^]^ Therefore, it is of great importance to develop a novel treatment method to inhibit mitochondrial autophagy and overcome the aforementioned disadvantages.

In cancer treatment, nanoparticles are characterized by low toxicity and high efficiency. They can specifically target cancer cells and effectively cross cell membranes through their controllable characteristics and size advantage.^[^ ^9^ ^]^ However, most nanoparticles entering cells will be trapped by lysosomes, and subsequently destroyed by the lysosomal enzymes.^[^ ^2b^ ^]^ Lysosomes significantly impact drug delivery efficiency and promote drug resistance.^[^ ^10^ ^]^ Consequently, the low intracellular delivery efficiency remains a major problem in nanomedicine.^[^ ^11^ ^]^ However, the accumulation of large inert substances and the overproduction of reactive oxygen species (ROS) in lysosomes can lead to lysosomal damage, thereby affecting their function and facilitating the escape of nanoparticles from lysosomes.^[^ ^12^ ^]^ In this context, the utilization of the self‐assembly strategy offers a promising approach to address the issue. Self‐assembly is a spontaneous process in which small molecular structures are assembled into ordered structures without human intervention. Nanoparticles with self‐assembly properties can effectively increase tumor cytotoxicity, reduce toxic side effects, and contribute to the long‐term retention of nanoparticles at the tumor site. Notably, the self‐assembly nanoparticles can influence the homeostasis of the surrounding environment, which is vital for promoting lysosomal escape.^[^ ^13^ ^]^ Nevertheless, it is still challenging to manipulate the intracellular specific and controllable assembly.^[^ ^14^ ^]^

**Scheme 1:**
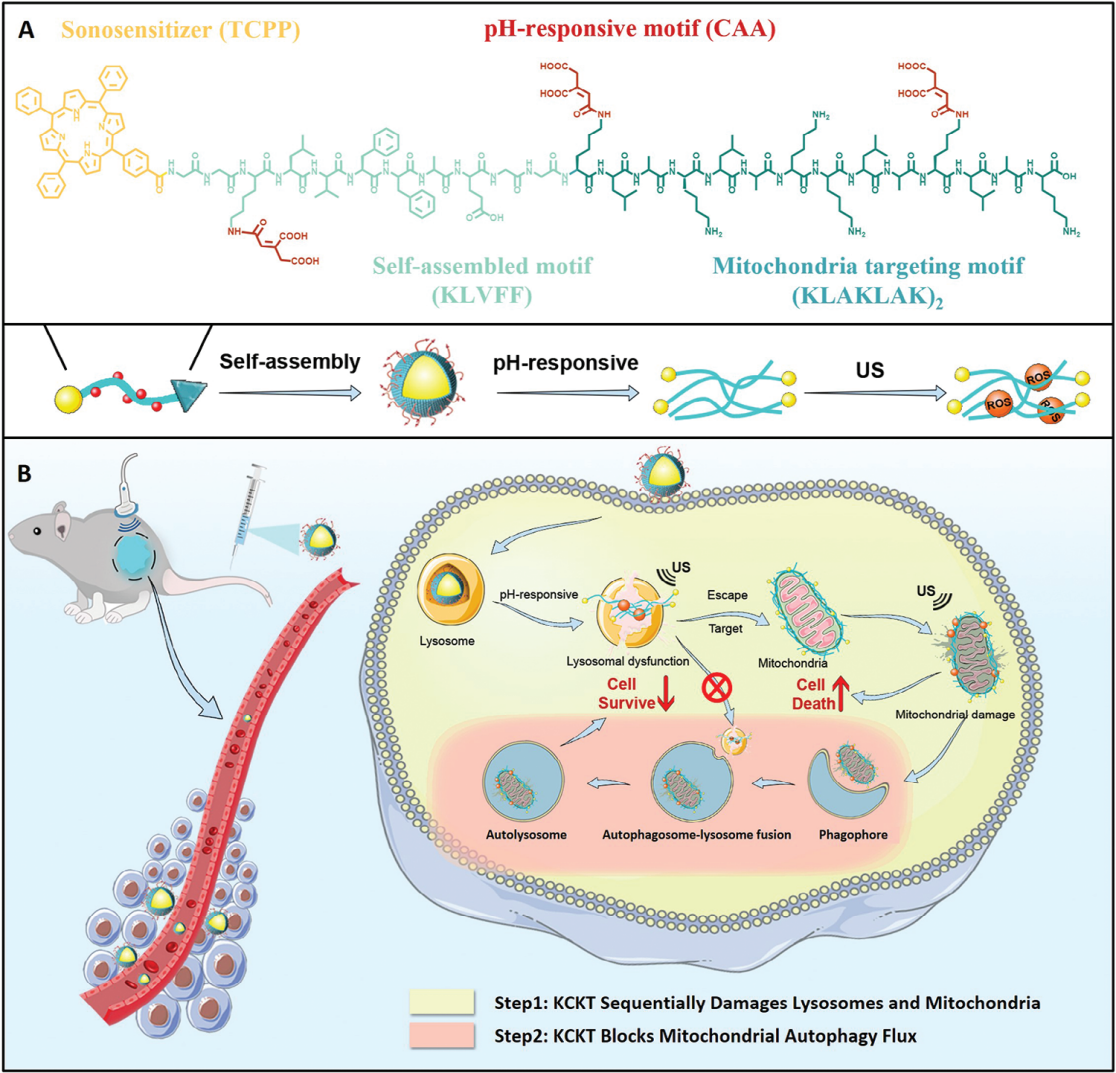
Schematic illustration of KCKT inhibiting tumor progression. A) Molecular structure of **KCKT** with pH‐responsiveness, self‐assembly, and sonodynamic therapy (SDT) function. B) The workflow of **KCKT** improves lysosomal escape, damages mitochondrion, and inhibits the autophagy flux. Step 1: **KCKT** sequentially damages lysosomes and mitochondria through the nanofiber transformation and SDT. Step 2: **KCKT** blocks mitochondrial autophagy flux by inhibiting lysosomal function.

Here, we developed an in situ transformable nanoparticle with pH responsive and nanofiber transformable capabilities named **KCKT**, which includes four parts: i) mitochondria targeting motif (KLAKLAK)_2_ for targeting mitochondria; ii) cis‐aconitic anhydride (CAA), serving as a pH‐responsive switch to trigger the transformation of self‐assembled peptide in the acidic environment; iii) KLVFF (Lys‐Leu‐Val‐Phe‐Phe, Aβ_16‐20_), a self‐assembled motif originating from β‐amyloid to form nanofibrous structure under stimulation;^[^ ^15^ ^]^ iv) 5‐(4‐carboxyphenyl)‐10,15,20‐triphenylporphyrin (TCPP), a sonosensitizer for sonodynamic therapy (SDT) (**Scheme** **1**a).^[^ ^16^ ^]^ Notably, CAA shares the same aspartic acid residue structure as RGD (Arg‐Gly‐Asp) peptides, thus possessing a similar targeting capability to the integrin α_v_β_3_, which is known to be overexpressed in bladder cancer.^[^ ^17^ ^]^ Consequently, **KCKT** can specifically target bladder cancer cells and be internalized into the lysosomes. Subsequently, CAA is hydrolyzed due to the acidic environment, triggering self‐assembly behaviors in situ and forming nanofiber structures. Simultaneously, the nanofibers damage lysosomal function and promote their escape from lysosomes. Then, generated ROS under ultrasound (US) further facilitates this process.^[^ ^18^ ^]^ Subsequently, the escaped nanofibers target mitochondria and retain them for the long term, which disrupts mitochondrial homeostasis and produces ROS, ultimately inducing cell apoptosis. More importantly, protective mitochondrial autophagy in cancer cells is significantly inhibited due to the functional damage of lysosomes, resulting in impaired mitochondria that cannot be degraded timely. Consequently, damaged mitochondria cannot efficiently produce adenosine triphosphate (ATP), leading to the overproduction of superoxide and further causing an imbalance in redox homeostasis. (Scheme 1b).^[^ ^19^ ^]^ In conclusion, our study increases drug delivery efficiency by disrupting lysosomal function and directly damages mitochondria while blocking mitochondrial autophagy using the self‐assembly strategy combined with SDT, which offers a new path for drug delivery and mitochondria‐targeted therapy.

## 2 Results

### 2.1 Synthesis and Characterization of KCKT

Based on the solid‐phase peptide synthesis method,^[^ ^20^ ^]^ we first synthesized KKT, the pro‐molecule of **KCKT** without pH‐responsive ability, and its non‐transformable control peptide KKT‐C. The molecular masses of KKT and KKT‐C were analyzed using a liquid chromatograph‐mass spectrometer (LC‐MS), and the peptides were purified using high‐performance liquid chromatography (HPLC) to confirm their structures and purities (Figure S1A,B, Supporting Information). Subsequently, we attached the CAA molecules to the amino groups of KKT and KKT‐C, forming **KCKT** and **KCKT‐C**, respectively. The ^1^H NMR spectra indicated that the modification of CAA is successful (Figures S2 and S3, Supporting Information).

**Figure 1:**
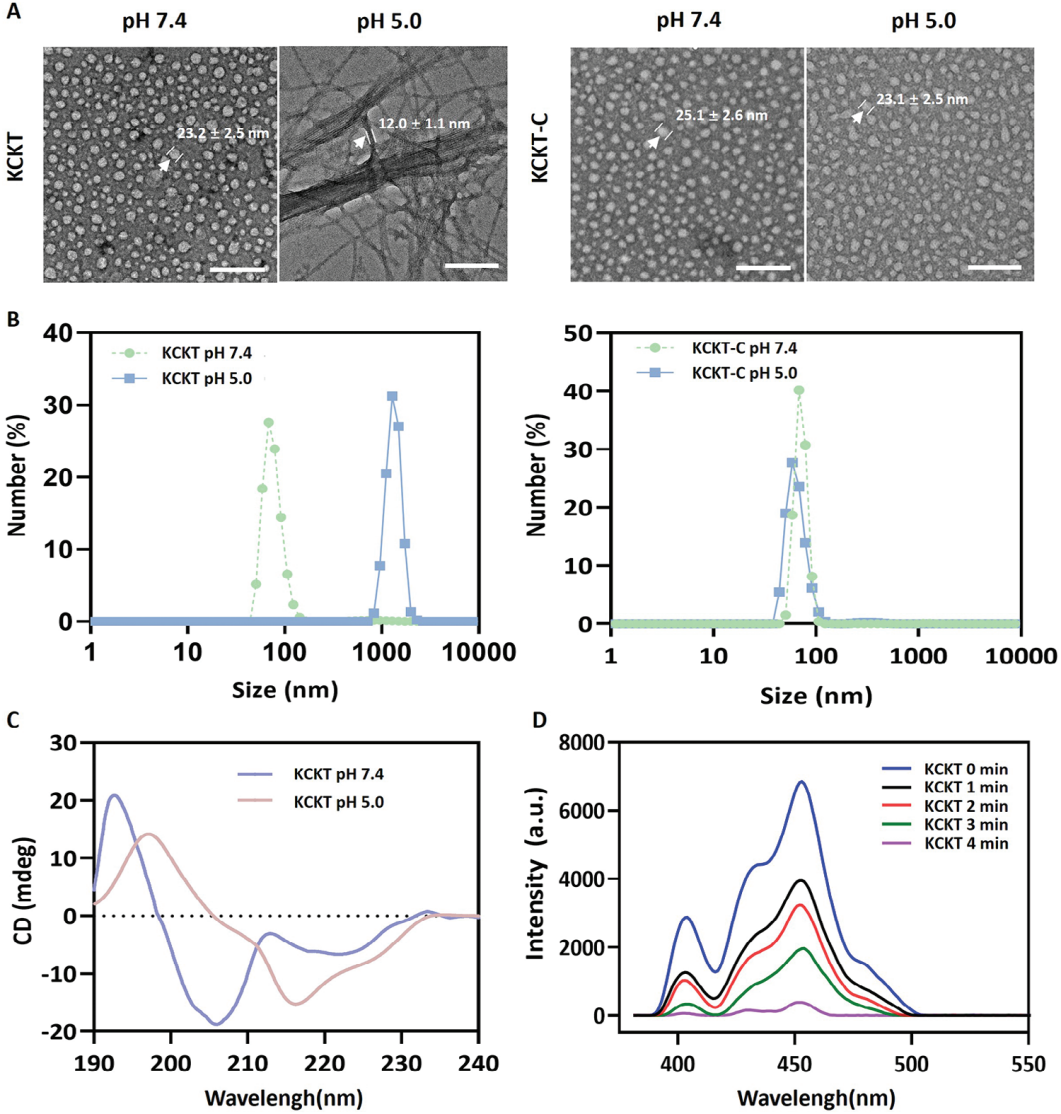
Physiochemical characterization of KCKT. A) Transmission electron microscopy (TEM) images of **KCKT** and **KCKT‐C** (5 × 10^−6^ m) at pH 7.4 and 5.0. Scale bar: 200 nm. B) Dynamic light scattering (DLS) spectra of **KCKT** and **KCKT‐C** in solution of pH 7.4 and 5.0. C) Circular dichroism (CD) spectra showing the secondary structure of **KCKT** at pH 7.4 and 5.0. D) The singlet oxygen (^1^O_2_) production of **KCKT** (5 × 10^−6^ m) after incubation with 9,10‐anthracenediyl‐bis(methylene)dimalonic acid (ABDA) under ultrasound (US) irradiation (1.25 W cm^−2^, 1.0 MHz).

Subsequently, we verified the characteristics of **KCKT** and **KCKT‐C** in different pH solutions. Transmission electron microscopy (TEM) was used to investigate the morphological changes. The results revealed that **KCKT** and **KCKT‐C** exhibited spherical structures with diameters of 23.2 ± 2.5 nm and 25.1 ± 2.6 nm at pH 7.4. Moreover, **KCKT** exhibited a significant nanofibrous transformation compared with the spherical structure of **KCKT‐C** under acidic conditions (pH 5.0) (**Figure** **1**A). The dynamic light scattering (DLS) results were consistent with TEM. Compared with the pH 7.4 solution, the particle size of KCKT increased significantly at pH 5.0 due to the aggregation of transformable nanofibers. Contrarily, KCKT‐C exhibited minimal change, further highlighting the influence of the acidic environment on the self‐assembly process (Figure 1B). Subsequently, the conformational changes in **KCKT** and **KCKT‐C** were analyzed by circular dichroism (CD) spectroscopy at various pH levels. The results indicated that **KCKT** had α‐helical structure at pH 7.4, and there were positive CD signals at 196 nm and negative signals at 216 nm at pH 5.0, implying the formation of nanofibers rich in β‐sheet structure. Meanwhile, **KCKT‐C** did not exhibit the typical β‐sheet characteristic peaks (Figure 1C; Figure S4A, Supporting Information). Furthermore, the zeta potential of **KCKT** was measured in solutions with different pH values. The results revealed that **KCKT** exhibited negative surface charges in a pH 7.4 solution due to the composition of CAA. However, the zeta potential increased from −11.5 mV to +2.9 mV at pH 5.0, suggesting CAA was hydrolyzed under acidic conditions (Figure S4B, Supporting Information). These results demonstrate that **KCKT** can be stimulated to transform in acidic environments.

Finally, we assessed the sonodynamic effects of **KCKT** and **KCKT‐C**. Briefly, 9, 10‐anthracene dimethylenedimalonic acid (ABDA), serving as an indicator for singlet oxygen (^1^O_2_), has a unique sensibility to ^1^O_2_. As illustrated in Figure 1D and Figure S4C (Supporting Information), under US irradiation (1.25 W cm^−2^, 1.0 MHz), the fluorescence intensity of ABDA gradually decreased with increasing reaction time in **KCKT** and **KCKT‐C** solutions, indicating the generation of ^1^O_2_.^[^ ^21^ ^]^ These results demonstrate that **KCKT** can effectively form nanofibrous structures in acidic environments and generate ^1^O_2_ under US irradiation.

### 2.2 Transformable KCKT Promotes Lysosomal Escape

**Figure 2:**
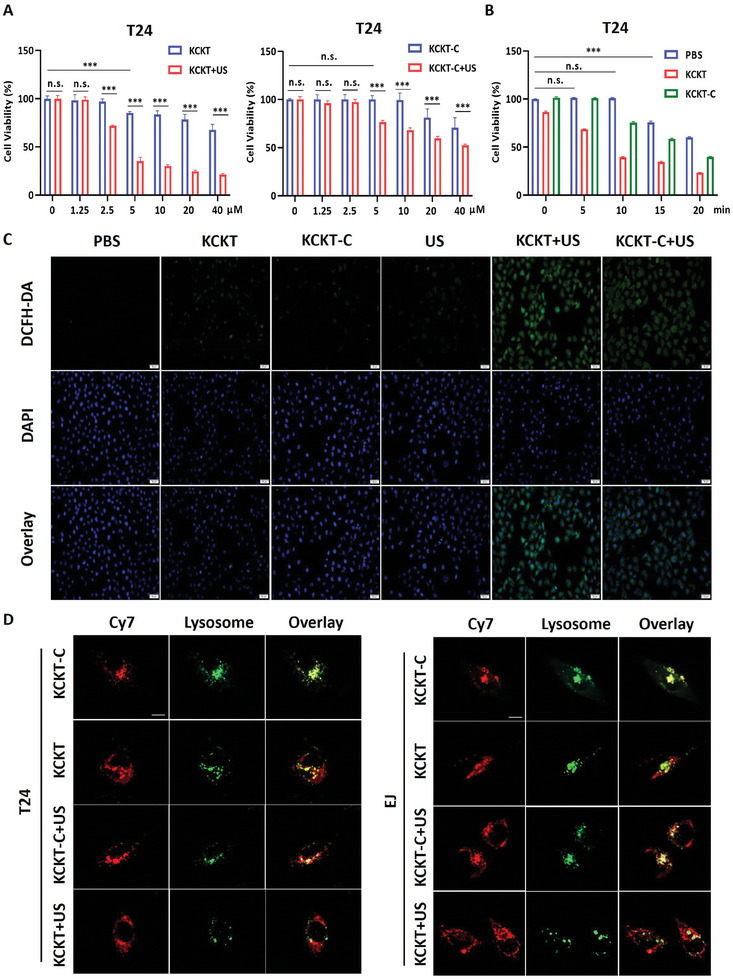
Transformable KCKT promotes lysosomal escape. A) Cell viability assay of T24 cells treated with different concentrations of **KCKT** or **KCKT‐C** in the presence or absence of US irradiation, *n* = 6. B) CCK‐8 assay of T24 cells treated with PBS, **KCKT**, or **KCKT‐C** (5 × 10^−6^ m) for varying US irradiation time, *n* = 6. C) Confocal fluorescence images showing intracellular reactive oxygen species (ROS) level with DCFH‐DA staining. Scale bar: 50 µm. D) The co‐localization images of nanoparticles and lysosomes in T24 and EJ cells treated with **KCKT**, **KCKT‐C**, **KCKT+US** and **KCKT‐C+US** (5 × 10^−6^ m), *n* = 3. Scale bar: 10 µm. Statistical analyses were performed using Student's t‐test A) and one‐way ANOVA with Bonferroni correction B). Data presented as mean ± SEM. ^***^ *p* < 0.001. n.s. means no significance. US: 1.25 W cm^−2^, 1.0 MHz.

Subsequently, we evaluated the bioactivity of **KCKT** and **KCKT‐C**. Initially, the viabilities of human bladder epithelial immortalized cells (SVHUC1) and bladder cancer cells (T24 and EJ) were examined at different concentrations of **KCKT** and **KCKT‐C**. The results indicated that **KCKT** and **KCKT‐C** did not affect the SVHUC1 cells viability until a concentration of 20 µM (Figure S5A, Supporting Information). Concurrently, T24 and EJ cells were inhibited in the **KCKT** solution at 5 µM, and the administration of SDT significantly enhanced cell suppression. Conversely, the addition of **KCKT‐C** alone did not reduce cell viability at the same dose (**Figure** **2**A; Figure S5B, Supporting Information). Subsequently, the efficacy of the SDT was assessed in vitro. The results demonstrated that US exposure for 10 min produced excellent outcomes, which led to cell death above 60% in the **KCKT+US** group, and non‐significant damage was observed in the PBS group. Meanwhile, in EJ and T24 cells, **KCKT‐C+US** treatment resulted in 24.7% ± 2.9% and 17.3% ± 2.5% cell death, respectively (Figure 2B; Figure S5C, Supporting Information). These findings suggest that the combination of the self‐assembly strategy with SDT can effectively enhance cancer cell death.

Subsequently, intracellular ROS were stained with 2,7‐dichloro‐dihydrofluorescein diacetate (DCFH‐DA) and investigated through a fluorescence microscope. Upon US irradiation, ROS levels were elevated in **KCKT+US** and **KCKT‐C+US** groups (Figure 2C; Figure S6A, Supporting Information). Simultaneously, the results were further confirmed using a fluorescence microplate, indicating that **KCKT** and **KCKT‐C** could generate ROS under US irradiation. Notably, **KCKT** presented a stronger ROS generation ability, probably due to its unique function on nanofiber formation (Figure S6B, Supporting Information). Lysosomal escape is pivotal for enhancing drug delivery efficiency, and previous studies have demonstrated that substance accumulation and ROS generation within lysosomes contribute to facilitating the process.^[^ ^12^ ^]^ Therefore, we detected the co‐localization fluorescence between nanoparticles labeled with cyanine 7 dye (Cy7) and lysosomes to monitor the escape capabilities of **KCKT** and **KCKT‐C** in T24 and EJ cells. The results indicated that the co‐localization coefficient of **KCKT‐C** with lysosomes in T24 cells was 0.94 ± 0.03, whereas the coefficient of the transformable **KCKT** was only 0.50 ± 0.06. This suggests that nanofiber transformation is sufficient to break through lysosomal barriers, thereby achieving intracellular delivery. We further examined the effects of combined US irradiation on lysosomal escape. After adding US irradiation, the co‐localization coefficient of **KCKT** with lysosome decreased to 0.22 ± 0.07, whereas the coefficient for **KCKT‐C** remained high at 0.69 ± 0.02. Similar results were observed in EJ cells, indicating that the combined treatment resulted in a more potent lysosomal escape effect (Figure 2D; Figure S7A, Supporting Information). Next, we observed a difference in the intracellular retention between **KCKT** and **KCKT‐C**. Fluorescence images revealed that **KCKT** could be maintained within the cells for at least 24 h. However, due to the lack of transformable ability, the fluorescence signal of **KCKT‐C** rapidly diminished within 12 h, highlighting the pivotal role of the self‐assembly strategy in long‐term retention (Figure S7B, Supporting Information).

### 2.3 Transformable KCKT Damages Mitochondrial Function and Induces Cell Apoptosis

**Figure 3:**
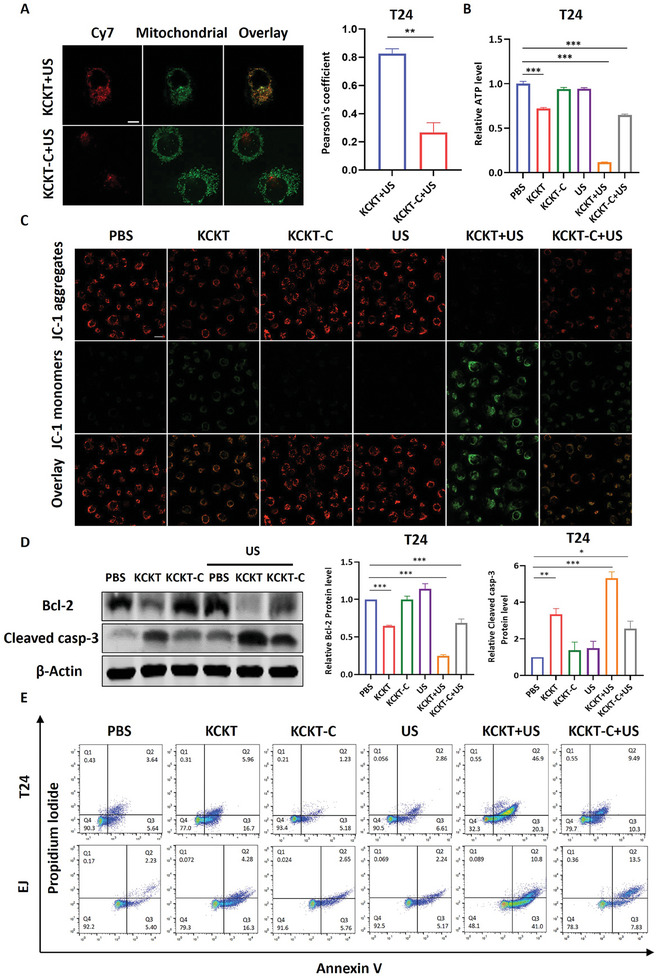
Transformable KCKT damages mitochondrial function and induces cell apoptosis. A) Confocal fluorescence images and co‐localization coefficient analysis showing the co‐localization between nanoparticles and mitochondria in T24 cells treated with **KCKT+US** or **KCKT‐C+US** (5 × 10^−6^ m). *n* = 3. Scale bar: 10 µm. B) The ATP levels of T24 cells in different treatments. *n* = 6. C) Mitochondrial membrane potentials of T24 cells treated with **KCKT**, **KCKT‐C**, US, **KCKT+US,** and **KCKT‐C+US**. Scale bar: 20 µm. D) Western blot analysis of Bcl‐2 and cleaved caspase‐3 in T24 cells treated with PBS, **KCKT**, **KCKT‐C**, US, **KCKT+US**, and **KCKT‐C+US** (5 × 10^−6^ m). *n* = 3. E) Flow cytometry analysis of apoptosis cells in different treatments. *n* = 3. Statistical analyses were performed using Student's t‐test (A) and one‐way ANOVA with Bonferroni correction (B, D). Data presented as mean ± SEM. ^***^ *p* < 0.001; ^**^ *p* < 0.01; ^*^ *p* < 0.05. US: 1.25 W cm^−2^, 1.0 MHz.

After the transformable **KCKT** successfully escaped from the lysosome, we detected its mitochondrial targeting ability. As revealed in **Figure** **3**A, the co‐localization fluorescence between **KCKT** and mitochondria demonstrated that the escaped **KCKT** could effectively target mitochondria in T24 cells. Contrarily, **KCKT‐C** presented poor co‐localization due to the failure of lysosomal escape. A similar observation was made in EJ cells (Figure S8A, Supporting Information). Next, we examined the effects of **KCKT** and **KCKT‐C** on mitochondrial function and apoptosis in T24 and EJ cells. Initially, we tested mitochondrial ATP production, and the results demonstrated that ATP levels in the **KCKT**, **KCKT+US**, and **KCKT‐C+US** groups were decreased compared to the PBS group. Notably, the attenuation of ATP production was more obvious in the **KCKT+US** group (Figure 3B; Figure S8B, Supporting Information). Subsequently, the mitochondrial membrane potential was determined using a mitochondrial membrane potential kit. Upon membrane potential decrease, JC‐1 became a monomer and emitted green fluorescence. With the addition of **KCKT**, **KCKT+US**, or **KCKT‐C+US**, the mitochondrial membrane exhibited green fluorescence, and **KCKT+US** exhibited the strongest fluorescence, indicating that mitochondrial function was severely impaired (Figure 3C; Figure S8C, Supporting Information).

Subsequently, we investigated whether the **KCKT** could trigger mitochondria apoptosis, which is generally considered to be caused by the explosive activation of caspase cascades.^[^ ^10^ ^]^ Therefore, we measured the expression of key mitochondrial apoptosis‐related factors, including active cleaved caspase3 and Bcl‐2. The results presented in Figure 3D demonstrated that the protein level of Bcl‐2 in the **KCKT**, **KCKT+US**, and **KCKT‐C+US** groups was significantly downregulated in T24 cells. Conversely, the expression of active cleaved caspase‐3 was upregulated compared to the PBS group, and the changes of proteins were more distinct in the **KCKT+US** group. These identical alterations were also observed in EJ cells, implying that **KCKT+US** treatment exhibits greater efficacy in inducing cellular death (Figure S9A, B, Supporting Information). Flow cytometry was used to directly quantify the levels of apoptosis. Our findings demonstrated that **KCKT** and **KCKT‐C+US** treatments induced cell death in varying degrees, but the efficiency of apoptosis was significantly lower than that of **KCKT+US** treatment in T24 and EJ cells (Figure 3E; Figure S9C, D, Supporting Information). Collectively, the transformable **KCKT** exhibits a greater capacity to promote mitochondrial damage compared to **KCKT‐C**, and the addition of US further enhances this effect.

### 2.4 Transformable KCKT Blocks Protective Autophagy by Impairing Lysosomal Function

**Figure 4:**
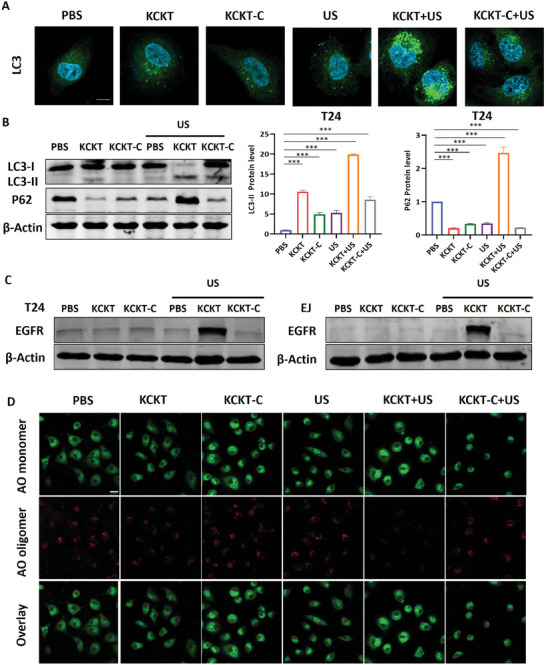
Transformable KCKT blocks protective autophagy by impairing lysosomal function. A) Representative images of immunofluorescence staining for LC3 in T24 cells treated with PBS, **KCKT**, **KCKT‐C**, US, **KCKT+US**, and **KCKT‐C+US** (5 × 10^−6^ m). Scale bar: 10 µm. B) Cellular expression of LC3 and P62 in T24 cells treated with various formulations. *n* = 3. C) Western blots showing the EGFR protein after incubation with EGF at 4 h. *n* = 3. D) Representative fluorescence images of AO‐stained T24 cells after various treatments. Scale bar: 20 µm. Statistical analyses were performed using one‐way ANOVA with Bonferroni correction. Data presented as mean ± SEM. ^***^ *p* < 0.001; ^**^ *p* < 0.01; ^*^ *p* < 0.05. US: 1.25 W cm^−2^, 1.0 MHz. n.s. means no significance.

Mitochondrial autophagy, as a protective mechanism in cancer, can specifically eliminate damaged mitochondria, which is a major obstacle in the therapeutic strategy of targeted mitochondria. Here, we aimed to investigate the autophagy activity affected by **KCKT** and **KCKT‐C** with or without US irradiation. First, LC3 expression, a typical autophagy marker, was verified by immunofluorescence staining in T24 and EJ cells. Normally, LC3 is dispersed in the cytoplasm. When autophagy occurs, LC3 accumulates on the autophagosome membrane to form bright fluorescent puncta under the fluorescence microscope.^[^ ^9^ ^]^ Our results revealed that LC3 fluorescence was evenly distributed in the PBS group. In the meantime, more puncta fluorescence of LC3 was observed in **KCKT**, **KCKT+US,** and **KCKT‐C+US** groups, respectively, and the **KCKT+US** group was particularly evident (**Figure** **4**A; Figure S10A, Supporting Information). In autophagy, cytoplasmic LC3‐I can be converted to membrane conjugated LC3‐II.^[^ ^22^ ^]^ Consequently, to further investigate the activation of autophagy, we evaluated the changes in LC3‐II levels using a western blot. Compared with the PBS group, the LC3‐II levels significantly increased under **KCKT**, **KCKT+US,** and **KCKT‐C+US** treatment. This phenomenon could be seen in autophagy flux activation or autophagy pathway blocking. Therefore, the P62 protein, a multifunctional ubiquitin‐binding protein preferentially degraded during autophagy activation, was selected for further analysis.^[^ ^8c^ ^]^ Figure 4B and Figure S10B (Supporting Information) illustrated that only **KCKT+US** treatment increased P62 protein levels compared to other groups, reflecting the blocking of autophagy flux. Simultaneously, the mRNA levels of LC3 and P62 were detected in T24 and EJ cells. The results demonstrated that neither **KCKT** and **KCKT‐C** treatment alone nor the combination with US could change their mRNA levels (Figure S10C,D, Supporting Information). These findings indicate that **KCKT+US** can effectively inhibit mitochondrial autophagy flux and destroy the self‐defense mechanism of cancer cells.

Subsequently, we revealed the reasons that the autophagy pathway was blocked. First, we examined the degradation capacity of lysosomes. EGFR is a cell membrane protein that can be degraded by lysosomes after binding with its ligand EGF.^[^ ^23^ ^]^ Hence, the EGFR degradation efficiency was measured after adding EGF in T24 and EJ cells. The results revealed that only the treatment with **KCKT+US** inhibited EGFR degradation, indicating the lysosomal degradation capacity was impaired (Figure 4C; Figure S11A, Supporting Information). We speculated that this might be due to nanofiber transformation or ROS generation alone being insufficient to completely damage lysosomes compared to the combination therapy. Next, we examined lysosomal acidification using acridine orange (AO) staining. As exhibited in Figure 4D and Figure S11B (Supporting Information), the red fluorescence of AO almost disappeared in the **KCKT+US** group, indicating that the lysosomal acidification was severely impaired. These results indicate that **KCKT** can effectively block mitochondrial autophagy flux by disrupting lysosomal function.

### 2.5 KCKT Promotes Cell Death in Patient‐Derived Tumor Organoids (PDO)

**Figure 5:**
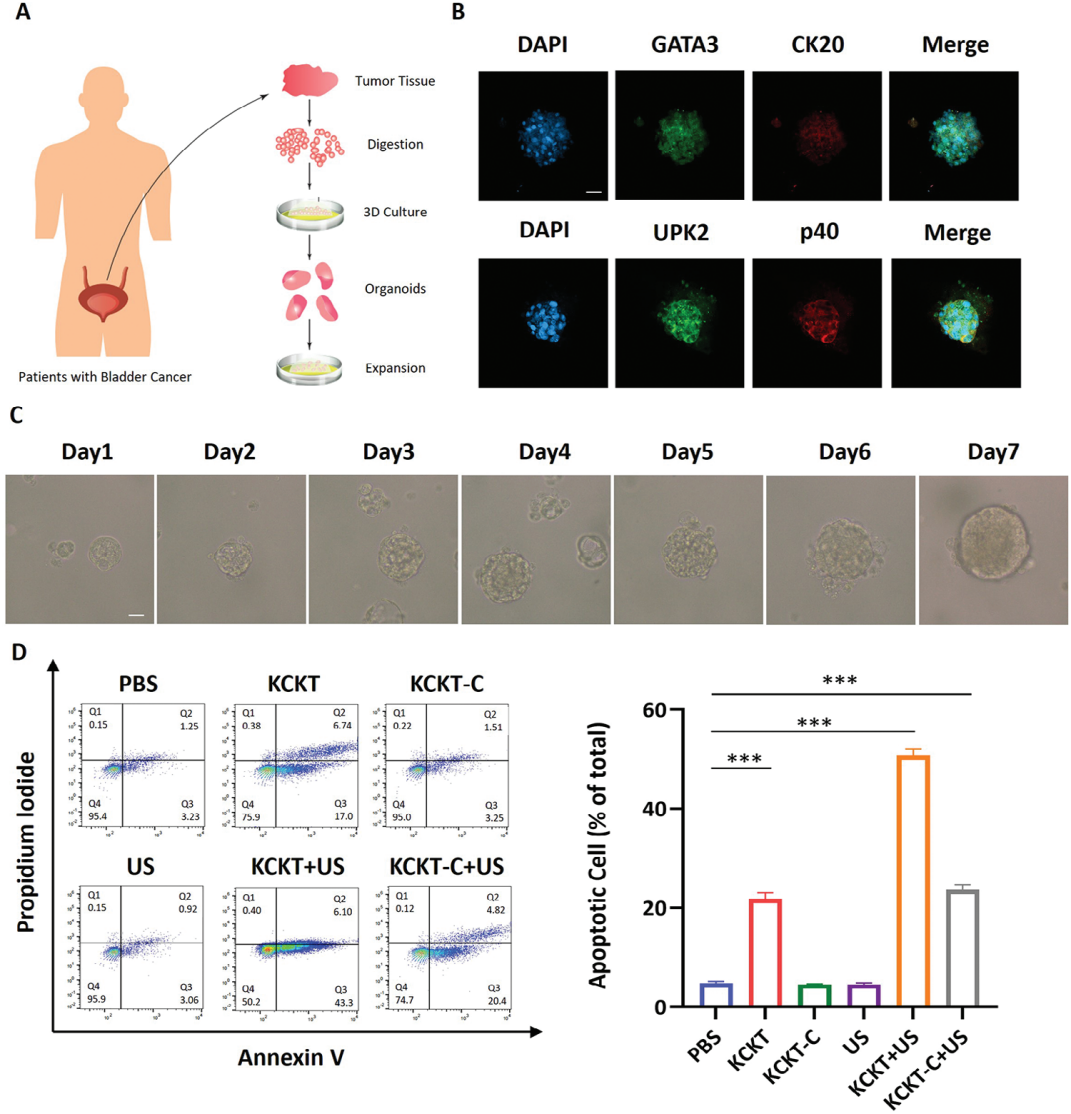
KCKT promotes cell death in PDO. A) Experimental design for bladder cancer tissue isolation and organoid culture. B) Immunostaining images of bladder cancer organoids biomarker. Scale bar: 50 µm. C) Representative images of bladder tumor organoids within the week. Scale bar: 50 µm. D) Flow cytometry and quantitative analysis of bladder cancer organoids treated with PBS, **KCKT**, **KCKT‐C**, US, **KCKT+US**, and **KCKT‐C+US** (5 × 10^−6^ m). *n* = 3. Statistical analyses were performed using one‐way ANOVA with Bonferroni correction. Data presented as mean ± SEM. ^***^ *p* < 0.001. US: 1.25 W cm^−2^, 1.0 MHz.

The heterogeneity exists in the tumor tissue of patients, so cell lines almost failed to simulate the tumor situation well.^[^ ^24^ ^]^ Thus, we constructed the PDO of patients with bladder cancer, which can more closely simulate cancer heterogeneity and biological behavior. **Figure** **5**A exhibits the bladder cancer tissue isolation and organoids culture process. The luminal marker GATA3, urothelial marker CK20, urothelial carcinoma marker UPK2, and squamous epithelium marker p40 were identified, indicating that the PDO of human bladder cancer was successfully constructed (Figure 5B). Simultaneously, for the assessment of organoid growth, images of organoids were captured daily for a week (Figure 5C). Subsequently, PDO was used to investigate the suppressive effects of nanoparticles. These experimental results were consistent with previous data from bladder cancer cell lines, indicating that **KCKT**, **KCKT‐C+US,** and **KCKT+US** promoted cell death. The combined application of **KCKT** and US had the most significant cytotoxic effects (Figure 5D).

### 2.6 KCKT Effectively Inhibits Tumor Progression In Vivo

**Figure 6:**
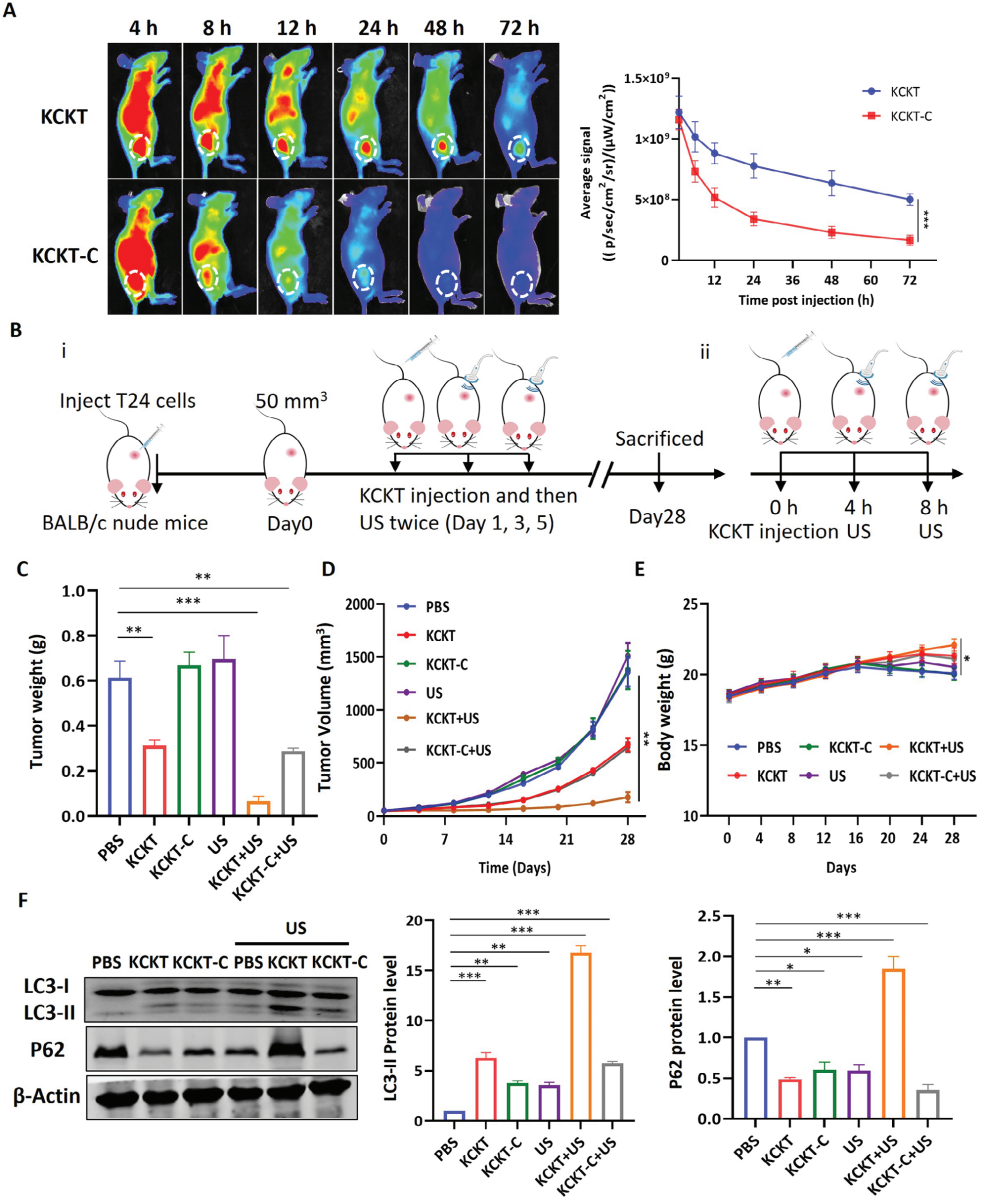
KCKT effectively inhibits tumor progression in vivo. A) Representative fluorescence images and quantitative analysis of mice injected with **KCKT** or **KCKT‐C** (200 µL, 100 × 10^−6^ m). *n* = 6. B) Schematic illustration of the antitumor treatment process in mice. (i) Mice were intravenously administered and then US irradiation for three doses. (ii) the single dose procedure. C) Tumor weight, D) tumor volume, and E) baby weight of mice treated with PBS, **KCKT**, **KCKT‐C**, US, **KCKT+US**, and **KCKT‐C+US**. *n* = 6. F) Western blot images and quantitative analysis of LC3 and P62 in the tumor tissue. *n* = 3. Statistical analyses were performed using one‐way ANOVA with Bonferroni correction. Data presented as mean ± SEM. ^***^ *p* < 0.001; ^**^ *p* < 0.01; ^*^ *p* < 0.05. US: 1.25 W cm^−2^, 1.0 MHz.

To further investigate the targeting capabilities of **KCKT** and **KCKT‐C** in vivo, we established a mice model by subcutaneous injection of T24 cells. Subsequently, **KCKT** and **KCKT‐C** were administered intravenously, and the fluorescence signal was captured using an in vivo imaging system (IVIS). **Figure** **6**A illustrated that **KCKT** and **KCKT‐C** accumulated at the tumor site within the first 4 h post‐injection. Following this, the **KCKT‐C** group exhibited a rapid decline in signal intensity, while the **KCKT** group maintained strong and sustained signals for at least 72 h. The mice were then sacrificed to detect the biological distribution of **KCKT** and **KCKT‐C** in the major organs. Notably, the fluorescence signals in tumors treated with **KCKT** were 3.4 times stronger than that in mice injected with **KCKT‐C** (Figure S12A, B, Supporting Information). These results suggest that nanofibers formation likely contributes to the long‐term retention of nanoparticles at the tumor site.

Subsequently, we evaluated the antitumor effect of **KCKT** and **KCKT‐C** in vivo as depicted in Figure 6B. **KCKT** and **KCKT‐C** were intravenously administered every 48 h for a total of three doses when the subcutaneous tumor reached 50 mm^3^. Additionally, US treatments were performed at 4 and 8 h after each injection. The mice were sacrificed for further studies after 28 days (Figure S13, Supporting Information). The results revealed that **KCKT** and **KCKT‐C+US** treatment reduced tumor weight by 49% ± 5.8% and 53% ± 3.1%, respectively, and the tumor inhibition ratio reached 89% ± 5.2% after treatment with **KCKT+US** (Figure 6C). Similarly, the tumor volume exhibited a comparable trend, suggesting that the combination treatment resulted in a more significant inhibitory effect (Figure 6D). Furthermore, the efficacy was evaluated by H&E staining and TUNEL assay. The tumor cells in the KCKT+US group exhibited more morphological changes and nuclear condensation compared to other groups. Additionally, the TUNEL staining demonstrated the strongest red fluorescence, indicating a higher level of apoptosis in the KCKT+US group. (Figure S14, Supporting Information). Next, we evaluated the toxic effects of **KCKT** and **KCKT**‐**C** in the experimental models. The body weights of the mice were monitored throughout the study. The results exhibited a non‐significant change during the initial treatment. The fluctuation during the later treatment period may be attributed to the development of cachexia caused by the tumor (Figure 6E). Additionally, hematoxylin and eosin (H&E) staining of the major organs, including the heart, liver, spleen, lung, and kidney, revealed insignificant organ damage and no unusual biochemical changes in biochemical analyses, implying good biosafety of the nanoparticles (Figure S15, Supporting Information). Finally, to determine the effect of **KCKT** treatment on autophagy in vivo, we analyzed LC3 and P62 expression changes in the tumor tissues (Figure 6F). The results were consistent with those observed in bladder cancer cell lines, indicating that **KCKT+US** could effectively block autophagy flux.

## 3 Conclusion

Herein, we designed an in situ transformable nanoparticle named **KCKT**, which can specifically target tumor sites and self‐assemble into nanofibrous structures within lysosomes by pH‐response. The structures alter the intracellular distribution of nanoparticles and facilitate lysosomal escape when combined with SDT therapy. Ultimately, the escaped **KCKT** effectively damages mitochondria and blocks mitochondrial autophagy by disrupting lysosomal function. Compared to existing mitochondrial targeted therapy strategies, this work utilizes a self‐assembly strategy to improve the efficiency of intracellular drug delivery and mitigate the influence of mitochondrial autophagy on mitochondrial therapy, breaking the conundrum that autophagy inhibitors cannot accurately target tumors. Therefore, **KCKT** demonstrates significant potential for improving the efficacy of cancer treatment.

## 4 Experimental Section

### Materials

All reagents used in the study were obtained from commercially available sources. The SVHUC1, T24, and EJ cell lines were purchased from the Cell Culture Center of the Institute of Basic Medical Sciences, Chinese Academy of Medical Sciences (Beijing, China). Glutamax, charcoal‐stripped fetal bovine serum (CS‐FBS), cell culture medium, and fetal bovine serum (FBS) were obtained from Gibco (Waltham, USA). The cell counting kit‐8 assay (CCK‐8), Mito‐Tracker Green, and Mitochondrial membrane potential assay kit (JC‐1) were purchased from the Beyotime Institute of Biotechnology (Shanghai, China). DCFH‐DA was acquired from Glpbio (Montclair, USA). Lyso Tracker Green DNA‐26 was sourced from Maokang Biotechnology (Shanghai, China). Epidermal growth factor (EGF), Matrigel, and collagenase were purchased from Coning Company (NY, USA). Y‐27632, TrypLE Express, and Dispase were obtained from STEMCELL Technologies (Vancouver, Canada). Female BALB/c mice were purchased from Vital River Laboratory Animal Technology Co., Ltd. (Beijing, China).

The following antibodies were used: LC3 (14600‐1‐AP, Proteintech Group, USA); P62 (18420‐1‐AP, Proteintech Group, USA); LAMP1 (67300‐1‐Ig, Proteintech Group, USA); EGFR (66455‐1‐Ig, Proteintech Group, USA); Bcl‐2 (12789‐1‐AP, Proteintech Group, USA); Cleaved Caspase‐3 (25128‐1‐AP, Proteintech Group, USA); β‐Actin (20536‐1‐AP, Proteintech Group, USA); GATA3 (66400‐1‐Ig, Proteintech Group, USA); CK20 (17329‐1‐AP, Proteintech Group, USA); UPK2 (21149‐1‐AP, Proteintech Group, USA); P40 (15105‐1‐AP, Proteintech Group, USA).

### Synthesis of KKT and KKT‐C Peptides

KKT and KKT‐C, the pro‐molecules of **KCKT** and **KCKT‐C** (non‐transformable control peptide) without cis‐aconitic anhydride (CAA) structure, were synthesized by solid‐phase synthesis method. Then, the KKT and KKT‐C were purified by reverse phase high‐performance liquid chromatography (HPLC) and analyzed by Liquid Chromatograph‐Mass Spectrometer (LC‐MS).

### Synthesis of KCKT and KCKT‐C peptides

The peptides KKT (10 mg) and CAA (6 mg) were dissolved in DMSO (1 mL), respectively. Then, the peptide solution was added to the CAA solution and reacted at room temperature for 2 h after complete dissolution. The peptides were washed three times with ether: ethanol = ratio of 5:2. Finally, H^1^‐NMR spectra were performed by dissolving with DMSO‐d^6^. The peptide KKT‐C was synthesized similarly to the experimental procedure described above.

### Characteristics of KCKT and KCKT‐C

The size distribution of **KCKT** and **KCKT‐C** under different solution conditions was detected by dynamic light scattering (DLS) with a detection angle of 90° (Zetasizer Nano ZS, Malvern, America). Additionally, the conformational changes were recorded by a Circular Dichroism Spectrometer (JASCO J 1500, Japan).

### Transmission Electron Microscope (TEM)

The peptides **KCKT** and **KCKT‐C** were incubated for 1 h in solutions with pH levels of 5.0 and 7.4, respectively. The excess liquid was removed using filter paper. Subsequently, uranyl acetate solution (10 µL) was applied onto a copper mesh to stain the samples for 3 min. After removing most of the staining solution with filter paper, the samples were imaged using a real‐time CCD imaging transmission electron microscope (HT7700 TEM).

### Determination of Reactive Oxygen Species (ROS) Production

5 mL ABDA (80 µM) solution was added to 5 mL **KCKT** or **KCKT‐C**. The mixture was placed in the dark for 30 min. 600 µL mixture was taken every 1 min for 5 min of US irradiation (1.0 MHz, 1.25 W cm^−2^) durations. The mixture was centrifuged for 3 min. The supernatant was taken to measure its fluorescence. The excitation wavelength was 365 nm and the emission wavelength was 380–600 nm.

### Sources of Patient Tissue Samples

All clinical samples were collected from patients undergoing radical cystectomy. All patients signed an informed consent form, and the study was approved by the Ethics Review Committee of the Fourth Hospital of Harbin Medical University (2021‐WZYSLLSC‐28).

### Establishment of Tumor Organoids

Fresh bladder cancer samples were collected from bladder cancer patients. These tissue samples were stored in ice and transported directly to the laboratory after being placed in media with 10 ng mL^−1^ EGF, 5% CS‐FBS, and 100 mg mL^−1^ Primocin. Subsequently, the tissues were cut into 0.2 mm pieces after being washed with media supplemented with 10 ng mL^−1^ EGF, 5% CS‐FBS, and 10 mM Y‐27632. The collagenase II containing Y‐27632 was used further to dissociate tissue for 15 min at 37 °C. The dissociated tissues were centrifuged at 350 g for 5 min and resuspended in DPBS. Consequently, the samples were centrifuged again and resuspended in 5 mL of TrypLE Express with Y‐27632 for another 5 min. Hank's balanced salt solution with 1% Glutamax, 1% HEPES, and 20% FBS was added to stop the digestion and further centrifugation. The resuspended solution was subjected to double filtration using a 100 mm cell strainer in order to eliminate any remaining undigested cells. The Filtered cells were collected by centrifugation and resuspend in the organoid culture media with 50% Matrigel at a density of 5 × 10^5^ cells∙mL^−1^. A volume of 50 µL mixture was dropped into the 24‐well plate. Subsequently, the 24‐well plate was first placed for 10 min and then turned upside down to solidify Matrigel for another 10 min. Finally, the organoid medium was added to the 24‐well plate and replaced every 2–3 days for continuous organoid culture for at least five generations.

Tumor organoids can be passed after seven days. A volume of 1 mg mL^−1^ dispase was added to the culture medium and incubated for 30 min. Consequently, the organoids were centrifuged at 350 g for 5 min and washed with DPBS. The organoids were then passaged at a 1:2 or 1:3 ratio after incubating with TrypLE Express for 3 min.

### Confocal Laser Scanning Microscopy (CLSM) for Cellular Imaging

Cells were seeded in a confocal dish and incubated with **KCKT** and **KCKT‐C** (5 × 10^−6^ m) for 4 h at 37 °C to detect the fluorescence intensity and colocalization. Subsequently, the cells were irradiated with a US transducer (1.25 W cm^−2^, 1.0 MHz) for 10 min and observed under a confocal laser scanning microscope (Hitachi, Japan).

### CCK‐8 Assay and Flow Cytometry for Cell Viability

SVHUC1, T24, and EJ cells were used to evaluate cell viability via CCK8 assay. The cells were seeded in 96‐well plates at a density of 1 × 10^4^ cells mL^−1^ and cultured with different concentrations of **KCKT** and **KCKT‐C** for 4 h. Subsequently, the cells were washed three times with PBS and then US for 10 min (1.25 W cm^−2^, 1.0 MHz). The cells were cultured at 37 °C and 5% CO_2_ for 24 h. Fresh medium containing 90 µL of serum‐free medium and 10 µL of CCK‐8 solution was added to 96‐well plates and incubated for 4 h. Finally, a microplate reader (Epoch, BioTek, Vermont, USA) was used to measure the absorbance at 450 nm. Cell apoptosis was evaluated by using ANXA5/annexin V‐FITC and PI and detected by flow cytometry (NovoCyteTM, ACEA Biosciences Inc., CA, USA).

### Acridine Orange (AO) Staining

T24 and EJ cells were seeded in a confocal dish and incubated with **KCKT** and **KCKT‐C** for 4 h (5 × 10^−6^ m) at 37 °C and 5% CO_2_. Each dish was treated with US for 10 min (1.25 W cm^−2^, 1.0 MHz). A total of 5 µg mL^−1^ AO (Aladdin, China) was added to the medium and incubated for 20 min. Subsequently, the cells were washed with PBS three times, followed by adding a serum‐free medium to the cells. The fluorescence images of cells were obtained using a confocal laser scanning microscope (Hitachi, Japan).

### JC‐1 Assay

Cancer cells were seeded in a confocal dish and treated with nanoparticles and US. The JC‐1 solution was added to the culture medium for 20 min. The medium was removed, and the cells were washed with PBS three times. Finally, the mitochondria were examined using a confocal laser scanning microscope (Hitachi, Japan).

### RNA Extraction and qRT‐PCR

Total RNA was extracted from the cell lines using Trizol reagent (Invitrogen, USA). Subsequently, reverse transcription was performed using a Takara kit (NHK, Japan). The mRNA levels were analyzed on an ABI Prism 7500 Fast Thermocycler (Applied Biosystems) for a total of 40 cycles.

### Western Blot Analysis

The cells were lysed in RIPA buffer (Beyotime, China). A bicinchoninic acid (BCA) kit (Beyotime, China) was applied to analyze the protein concentration and then separated by sodium dodecyl sulfate‐polyacrylamide gel electrophoresis (SDS‐PAGE). The gel was blotted onto PVDF membranes (Millipore, USA). The membranes were blocked in a blocking buffer containing 5% nonfat milk and subsequently incubated with specific antibodies. Consequently, it was incubated with the corresponding secondary antibody and quantified using Odyssey v1.2 software (LI‐COR, USA).

### Analysis of EGFR Degradation

The cells were seeded in a culture dish and incubated with **KCKT** and **KCKT‐C** for 4 h (5 × 10^−6^ m) at 37 °C. Consequently, the cells were irradiated by the US transducer (1.25 W cm^−2^, 1.0 MHz) for 10 min. Subsequently, cells were incubated with a medium containing 100 ng mL^−1^ of EGF for 4 h at 37 °C. Finally, the total amount of protein in the cells was collected for western blot analysis.

### Intracellular ROS Measurement

The DCFH‐DA (Beyotime, China) staining was performed to measure cell ROS levels. The cells were incubated with **KCKT** and **KCKT‐C** for 4 h (5 × 10^−6^ m) at 37 °C and 5% CO_2_. The cells were washed with PBS. DCFH‐DA was added for 20 min, followed by US irradiation (1.25 W cm^−2^, 1.0 MHz). Cellular fluorescence was imaged using CLSM (Zeiss Axio‐Imager LSM‐800) and detected by the fluorescent microplate reader (Infinite 200 Pro, Tecan, Switzerland).

### In vivo tumor targeting and bio‐Distribution

All animal experiments were approved by the Committee for Animal Research of the Fourth Hospital of Harbin Medical University (2022‐DWSYLLCZ‐99). To assess the in vivo bio‐distribution of **KCKT** and **KCKT‐C**, experimental models were developed by subcutaneously injecting T24 cells (5 × 10^6^ cells in 200 µL of PBS) into the BALB/c nude mice. The **KCKT** and **KCKT‐C** were intravenously injected into the mice (200 µL, 100 × 10^−6^ m). The fluorescence signal was captured by a CRi Maestro 2 multispectral fluorescence small animal IVIS at different times. Then, the mice were sacrificed further to examine the biological distribution of the major organs.

### In vivo Antitumor Effect

The BALB/c nude mice were subcutaneously injected with T24 cells to establish a xenografts animal model. The mice were randomly divided into six groups and treated with PBS, **KCKT**, **KCKT‐C**, PBS+US, **KCKT+US**, or **KCKT‐C+US** (200 µL, 100 × 10^−6^ m) by intravenous injection every 48 h for 3 times when the tumor volume reached 50 mm^3^. The US (1.25 W cm^−2^, 1.0 MHz) was administered after being injected with **KCKT** and **KCKT‐C** for 4 and 8 h. Subsequently, the mice weights were measured every seven days during the experiment, and the mice were sacrificed to evaluate the antitumor effect at 28 days.

### Statistical Analysis

Prism 8 software (version 8.2.1) was used for statistical analysis. Data were expressed as the mean ± standard error of the mean (SEM), and each experiment was independently conducted at least three times. Two‐tailed unpaired Student's t‐test was utilized for comparing data between two groups, while One‐way ANOVA was employed for comparisons among multiple groups, with Bonferroni test correction. The sample size (n) was shown in the corresponding figure legends. P value less than 0.05 was considered statistically significant. ^***^ *p* < 0.001, ^**^ *p* < 0.01, ^*^ *p* < 0.05, n.s. means no significance.

## Conflict of Interest

The authors declare no conflict of interest.

## Supporting information



Supporting Information

## Data Availability

The data that support the findings of this study are available in the supplementary material of this article.

## References

[1] a) L. Wang , D. J. Klionsky , H. M. Shen , Nat. Rev. Mol. Cell Biol. 2023, 24, 186; 36097284 10.1038/s41580-022-00529-z

[2] a) S. Delaunay , G. Pascual , B. Feng , K. Klann , M. Behm , A. Hotz‐Wagenblatt , K. Richter , K. Zaoui , E. Herpel , C. Munch , S. Dietmann , J. Hess , S. A. Benitah , M. Frye , Nature 2022, 607, 593; 35768510 10.1038/s41586-022-04898-5 PMC9300468

[3] D. Zhang , D. Man , J. Lu , Y. Jiang , B. Ding , R. Su , R. Tong , J. Chen , B. Yang , S. Zheng , D. Chen , J. Wu , Adv. Sci. (Weinh) 2023, 10, e2206669. 36994647 10.1002/advs.202206669 PMC10214260

[4] K. Vasan , N. S. Chandel , J. Clin. Invest. 2024, 134, e176736. 38299592 10.1172/JCI176736 PMC10836798

[5] S. Wang , H. Long , L. Hou , B. Feng , Z. Ma , Y. Wu , Y. Zeng , J. Cai , D. W. Zhang , G. Zhao , Signal Transduct Target. Ther. 2023, 8, 304. 37582956 10.1038/s41392-023-01503-7 PMC10427715

[6] M. A. Eldeeb , R. A. Thomas , M. A. Ragheb , A. Fallahi , E. A. Fon , Physiol. Rev. 2022, 102, 1721. 35466694 10.1152/physrev.00041.2021

[7] K. Qiu , W. Zou , Z. Fang , Y. Wang , S. Bell , X. Zhang , Z. Tian , X. Xu , B. Ji , D. Li , T. Huang , J. Diao , ACS Nano 2023, 17, 4716. 36848459 10.1021/acsnano.2c11003

[8] a) H. Wang , H. Bai , J. Wang , X. Zhou , H. Chen , L. Wang , H. Ren , Z. Liu , W. Zhuo , Z. Zhou , J. Tang , Z. Li , J. Wang , Y. Shen , T. Zhou , X. Liu , Biomaterials 2022, 283, 121458; 35286855 10.1016/j.biomaterials.2022.121458

[9] M. Liu , Y. Feng , Y. Lu , R. Huang , Y. Zhang , Y. Zhao , R. Mo , Sci. Adv. 2024, 10, eadk2444. 38478602 10.1126/sciadv.adk2444 PMC10936870

[10] J. Yang , A. Griffin , Z. Qiang , J. Ren , Signal Transduct. Target Ther. 2022, 7, 379. 36402753 10.1038/s41392-022-01243-0 PMC9675787

[11] Y. Fu , F. Ye , X. Zhang , Y. He , X. Li , Y. Tang , J. Wang , D. Gao , ACS Nano 2022, 16, 18376. 36355037 10.1021/acsnano.2c06356

[12] a) M. Borkowska , M. Siek , D. V. Kolygina , Y. I. Sobolev , S. Lach , S. Kumar , Y. K. Cho , K. Kandere‐Grzybowska , B. A. Grzybowski , Nat. Nanotechnol. 2020, 15, 331; 32203435 10.1038/s41565-020-0643-3

[13] a) Z. Wang , H. W. An , D. Hou , M. Wang , X. Zeng , R. Zheng , L. Wang , K. Wang , H. Wang , W. Xu , Adv. Mater. 2019, 31, 1807175; 10.1002/adma.201807175 30663139

[14] J. Wang , L. Hu , H. Zhang , Y. Fang , T. Wang , H. Wang , Adv. Mater. 2022, 34, 2104704. 10.1002/adma.202104704 34632634

[15] L. Zhang , D. Jing , N. Jiang , T. Rojalin , C. M. Baehr , D. Zhang , W. Xiao , Y. Wu , Z. Cong , J. J. Li , Y. Li , L. Wang , K. S. Lam , Nat. Nanotechnol. 2020, 15, 145. 31988501 10.1038/s41565-019-0626-4 PMC7147967

[16] N. Singh , J. Kim , J. Kim , K. Lee , Z. Zunbul , I. Lee , E. Kim , S. G. Chi , J. S. Kim , Bioact. Mater. 2023, 21, 358. 36185736 10.1016/j.bioactmat.2022.08.016 PMC9483748

[17] a) Y. Cong , L. Ji , Y. J. Gao , F. H. Liu , D. B. Cheng , Z. Hu , Z. Y. Qiao , H. Wang , Angew. Chem. Int. Ed. Engl. 2019, 58, 4632; 30695128 10.1002/anie.201900135

[18] L. Wang , C. Li , J. Wang , G. Yang , Y. Lv , B. Fu , L. Jian , J. Ma , J. Yu , Z. Yang , P. Wu , G. Li , X. Liu , Z. Kang , Z. Wang , L. Wang , H. Wang , W. Xu , Adv. Mater. 2022, 34, 2203518. 10.1002/adma.202203518 36004775

[19] L. Cui , A. M. Gouw , E. L. LaGory , S. Guo , N. Attarwala , Y. Tang , J. Qi , Y. S. Chen , Z. Gao , K. M. Casey , A. A. Bazhin , M. Chen , L. Hu , J. Xie , M. Fang , C. Zhang , Q. Zhu , Z. Wang , A. J. Giaccia , S. S. Gambhir , W. Zhu , D. W. Felsher , M. D. Pegram , E. A. Goun , A. Le , J. Rao , Nat. Biotechnol. 2021, 39, 357. 33077961 10.1038/s41587-020-0707-9 PMC7956242

[20] N. Hartrampf , A. Saebi , M. Poskus , Z. P. Gates , A. J. Callahan , A. E. Cowfer , S. Hanna , S. Antilla , C. K. Schissel , A. J. Quartararo , X. Ye , A. J. Mijalis , M. D. Simon , A. Loas , S. Liu , C. Jessen , T. E. Nielsen , B. L. Pentelute , Science 2020, 368, 980. 32467387 10.1126/science.abb2491

[21] G. Xu , C. Li , C. Chi , L. Wu , Y. Sun , J. Zhao , X. H. Xia , S. Gou , Nat. Commun. 2022, 13, 3064. 35654794 10.1038/s41467-022-30721-w PMC9163081

[22] J. N. S. Vargas , M. Hamasaki , T. Kawabata , R. J. Youle , T. Yoshimori , Nat. Rev. Mol. Cell Biol. 2023, 24, 167. 36302887 10.1038/s41580-022-00542-2

[23] Q. Liu , Y. Luo , Y. Zhao , P. Xiang , J. Zhu , W. Jing , W. Jin , M. Chen , R. Tang , H. Yu , Bioact. Mater. 2022, 8, 478. 34541414 10.1016/j.bioactmat.2021.06.004 PMC8429627

[24] M. S. Esfahani , E. G. Hamilton , M. Mehrmohamadi , B. Y. Nabet , S. K. Alig , D. A. King , C. B. Steen , C. W. Macaulay , A. Schultz , M. C. Nesselbush , J. Soo , J. G. Schroers‐Martin , B. Chen , M. S. Binkley , H. Stehr , J. J. Chabon , B. J. Sworder , A. B. Hui , M. J. Frank , E. J. Moding , C. L. Liu , A. M. Newman , J. M. Isbell , C. M. Rudin , B. T. Li , D. M. Kurtz , M. Diehn , A. A. Alizadeh , Nat. Biotechnol. 2022, 40, 585. 35361996 10.1038/s41587-022-01222-4 PMC9337986

