# Supplementary material for: In Situ Transformable Nanoparticle Effectively Suppresses Bladder Cancer by Damaging Mitochondria and Blocking Mitochondrial Autophagy Flux
Source: Adv Sci (Weinh). 2024 Dec 9;12(5):2409425. doi: 10.1002/advs.202409425 (PMC11791963; doi:10.1002/advs.202409425)
Supplement: Supplementary file 1 — Supporting Information [file ADVS-12-2409425-s001.docx]

Supporting Information

In Situ Transformable Nanoparticle Effectively Suppresses Bladder Cancer by Damaging Mitochondria and Blocking Mitochondrial Autophagy Flux

Yulin Lv, Benli Song, Guang Yang, Yuting Wang, Zeyu Wu, Minggui Si, Zongzheng Yang, Huilin Chen, Chen Liu, Min Li, Yinshi Zhang, Zengying Qiao*, Lu Wang*, Wanhai Xu*


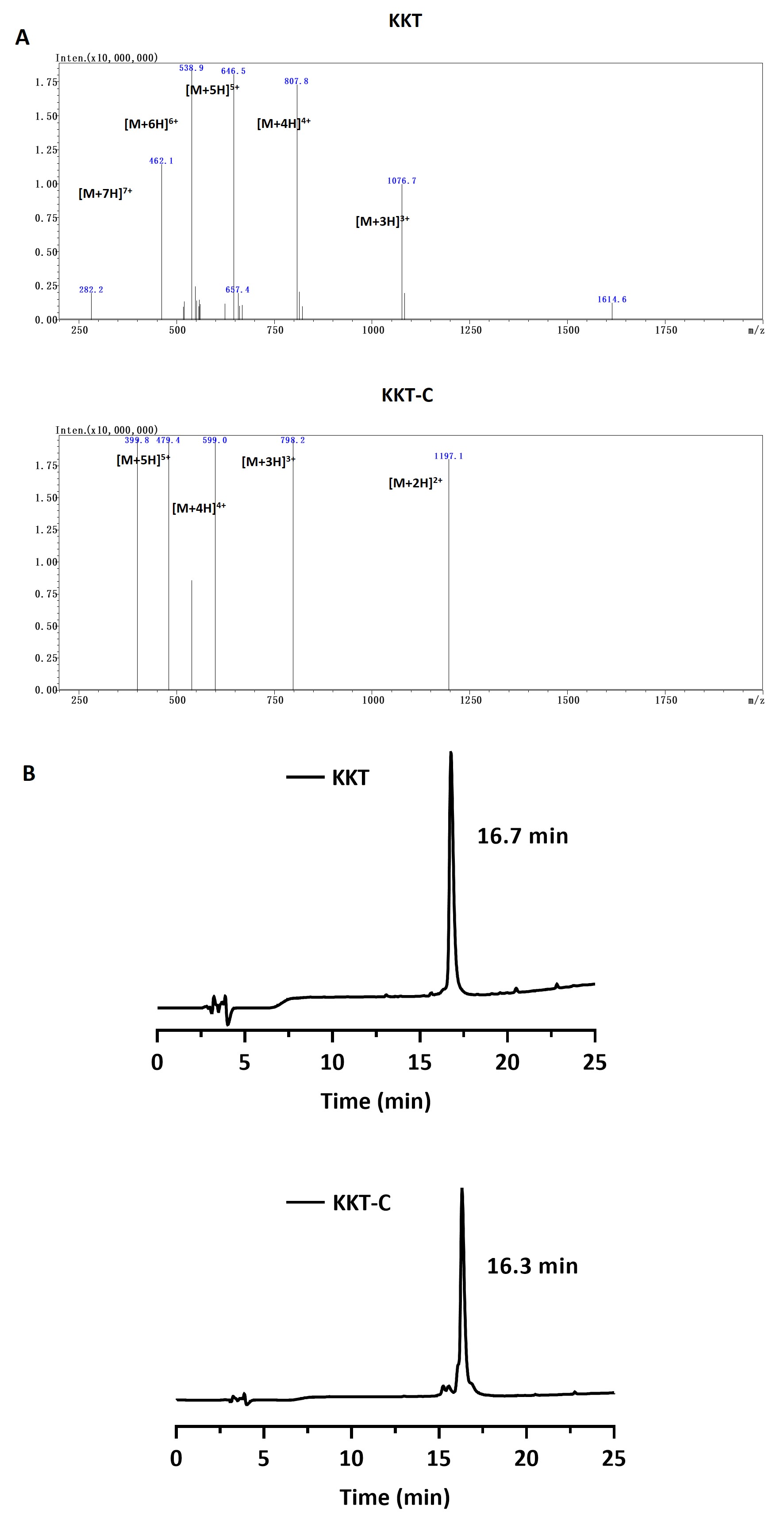


**Figure S1.** A) Liquid chromatograph-mass spectrometer (LC-MS) of **KKT** and **KKT-C**. B) The high-performance liquid chromatography (HPLC) analysis of **KKT** and **KKT-C**.


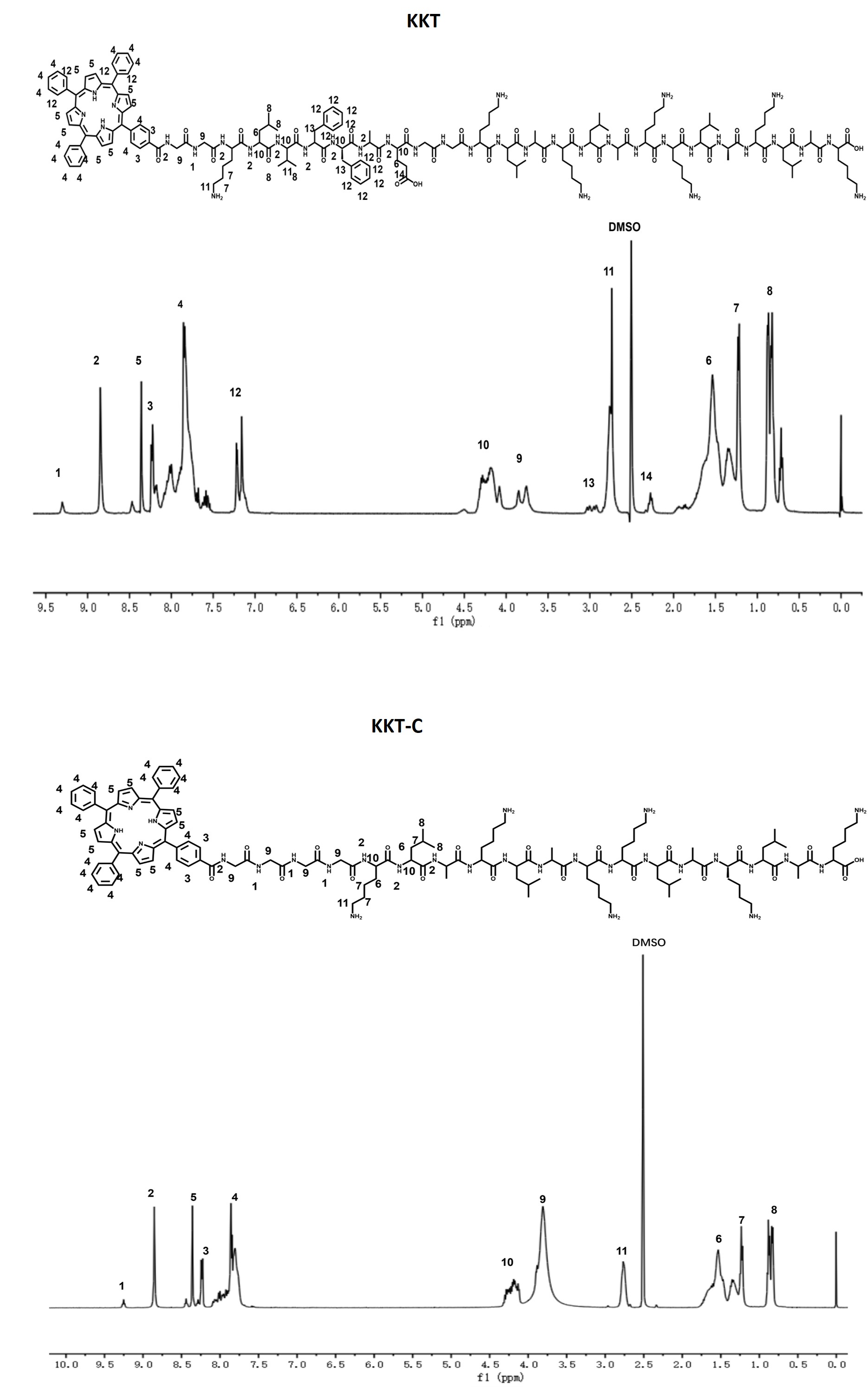


**Figure S2.** The ^1^H NMR spectra of **KKT** and **KKT-C** without CAA.


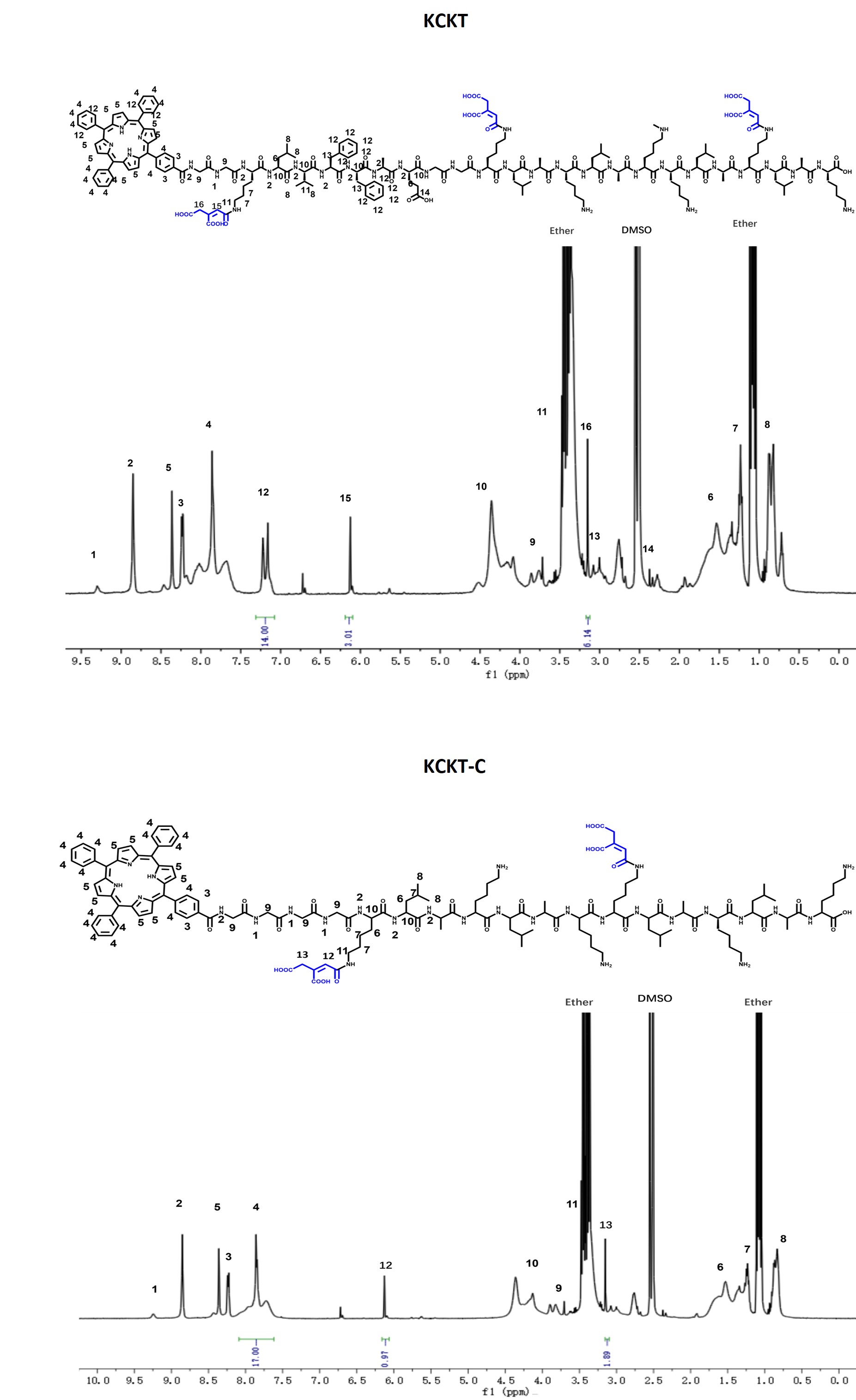


**Figure S3.** The ^1^H NMR spectra of **KCKT** and **KCKT-C** after connecting CAA.


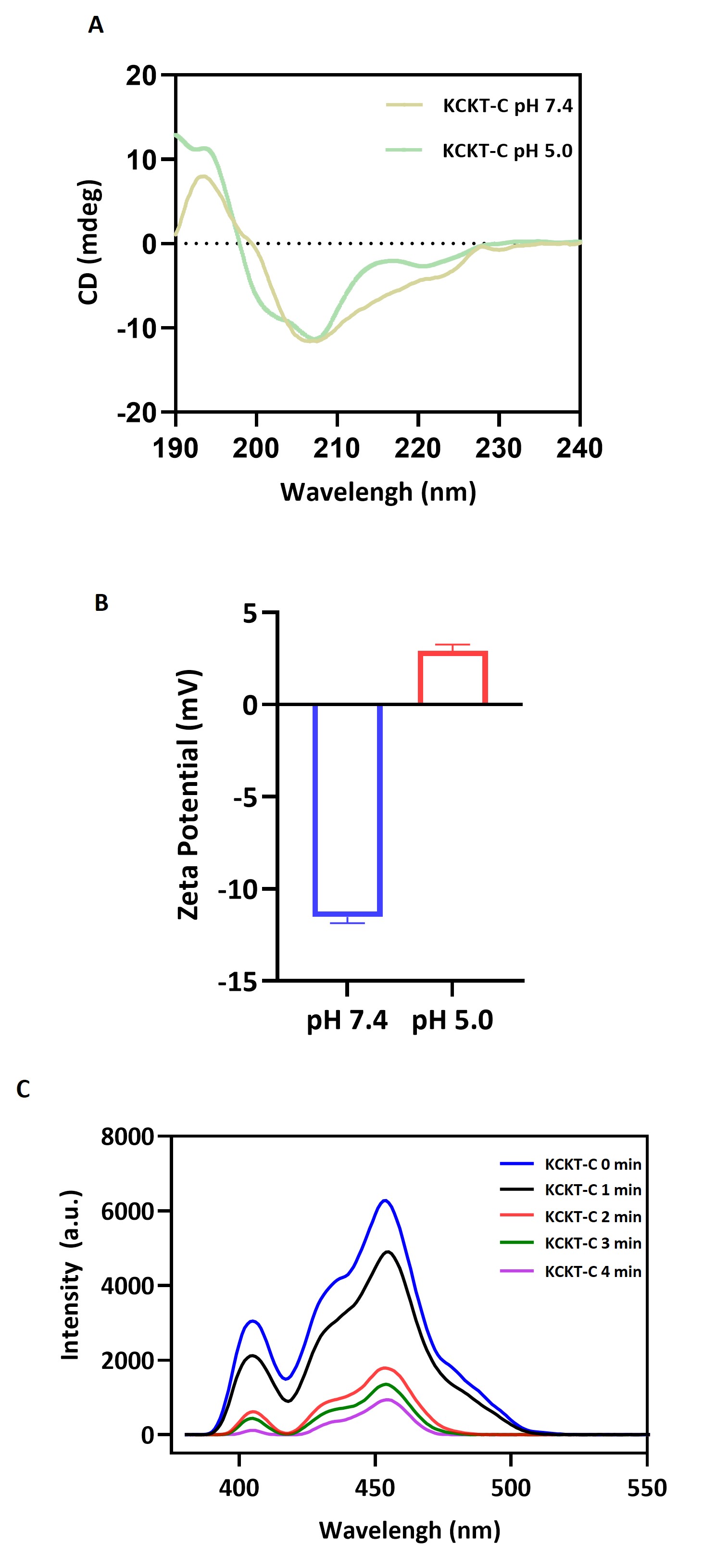


**Figure S4.** A) Circular dichroism (CD) spectra showing conformation change of **KCKT-C** at pH 7.4 and 5.0. B) The zeta potential of **KCKT** in different pH solution. C) The singlet oxygen (^1^O_2_) production of **KCKT-C** after incubation with 9,10-anthracenediyl-bis(methylene)dimalonic acid under ultrasound (US) irradiation (1.25 W cm^−2^, 1.0 MHz).


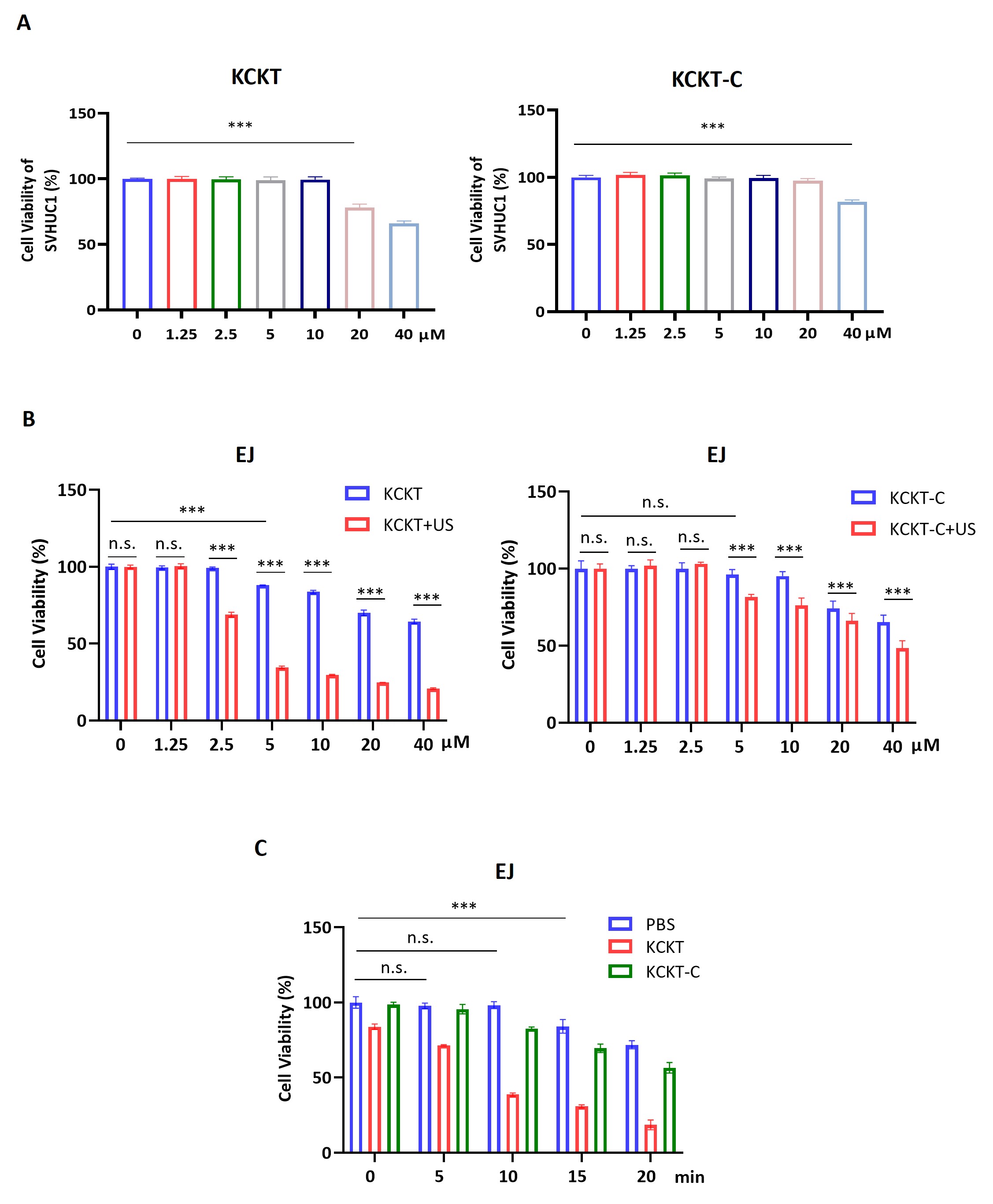


**Figure S5. Cytotoxicity of KCKT and KCKT-C against cells in vitro.** A) The viability of SVHUC1 cells treated with different concentrations of **KCKT** or **KCKT-C**, n = 6. B) CCK-8 assay of EJ cells after incubation with different concentrations of **KCKT** or **KCKT-C**, n = 6. C) The cell viability of EJ cells treated with **KCKT** or **KCKT-C** for varying US irradiation time, n = 6. Statistical analyses were performed using Student’s t-test (B) and one-way ANOVA with Bonferroni correction (A, C). Data presented as mean ± SEM. ***p < 0.001. n.s. means no significance. US: 1.25 W cm^−2^, 1.0 MHz.


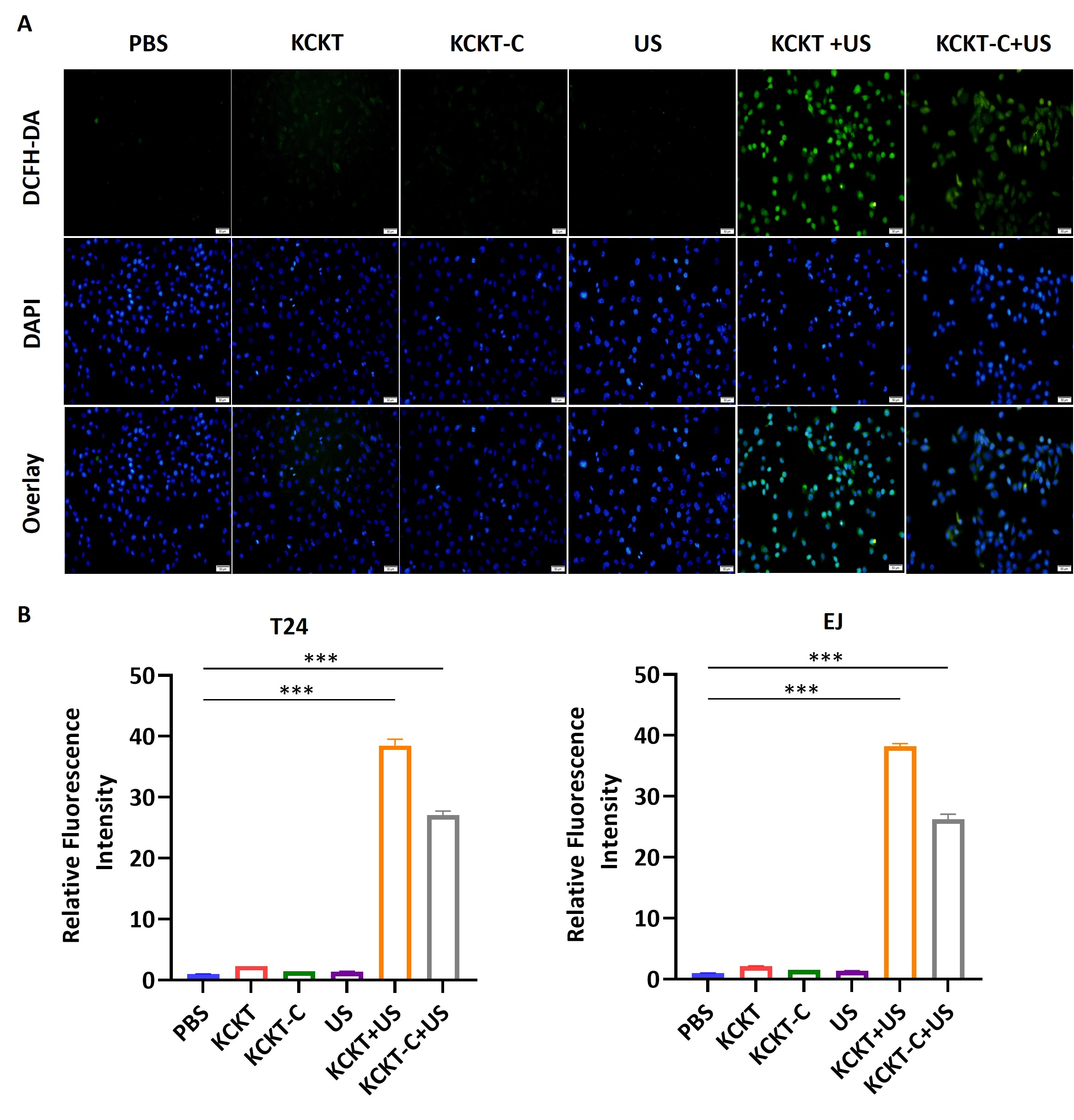


**Figure S6. ROS generated levels after US irradiation in cells.** A) Fluorescence images of EJ cells showing intracellular ROS production triggered by PBS, **KCKT**, **KCKT-C**, US, **KCKT+US**, and **KCKT-C+US** (5 × 10^−6^ M), Scale bar: 50 µm. B) The statistical analysis of intracellular ROS levels in T24 and EJ cells under fluorescence microplate. n = 6. Statistical analyses were performed using one-way ANOVA with Bonferroni correction. Data presented as mean ± SEM. ***p < 0.001. US: 1.25 W cm^−2^, 1.0 MHz.


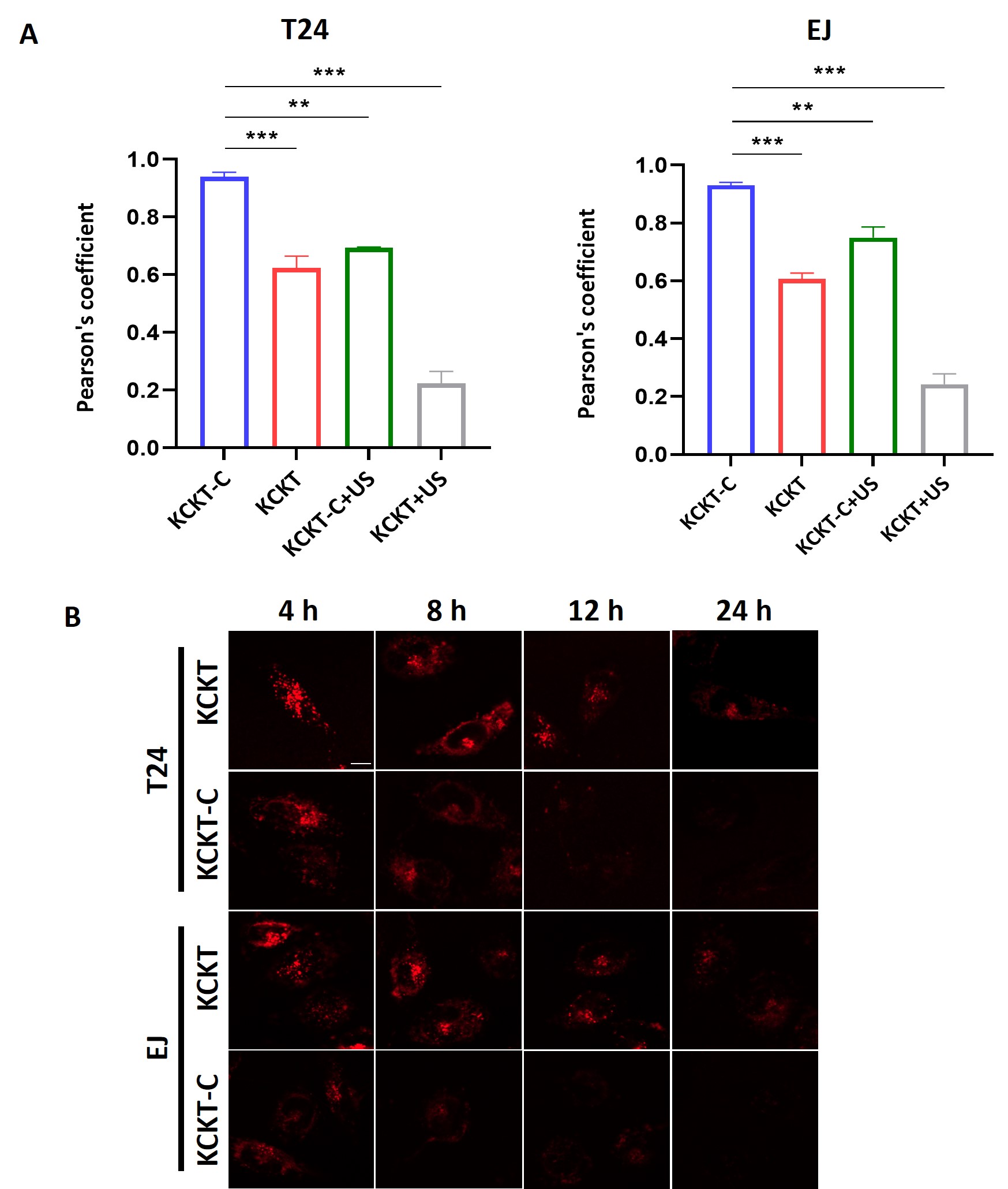


**Figure S7. Lysosomal escape and intracellular retention ability detection.** A) The analysis of the co-localization coefficient between nanoparticles and lysosomes. n = 3. B) The intracellular retention of **KCKT** or **KCKT-C** in T24 and EJ cells. Scale bar: 10 µm. Statistical analyses were performed using one-way ANOVA with Bonferroni correction. Data presented as mean ± SEM. ***p < 0.001; **p < 0.01. US: 1.25 W cm^−2^, 1.0 MHz.


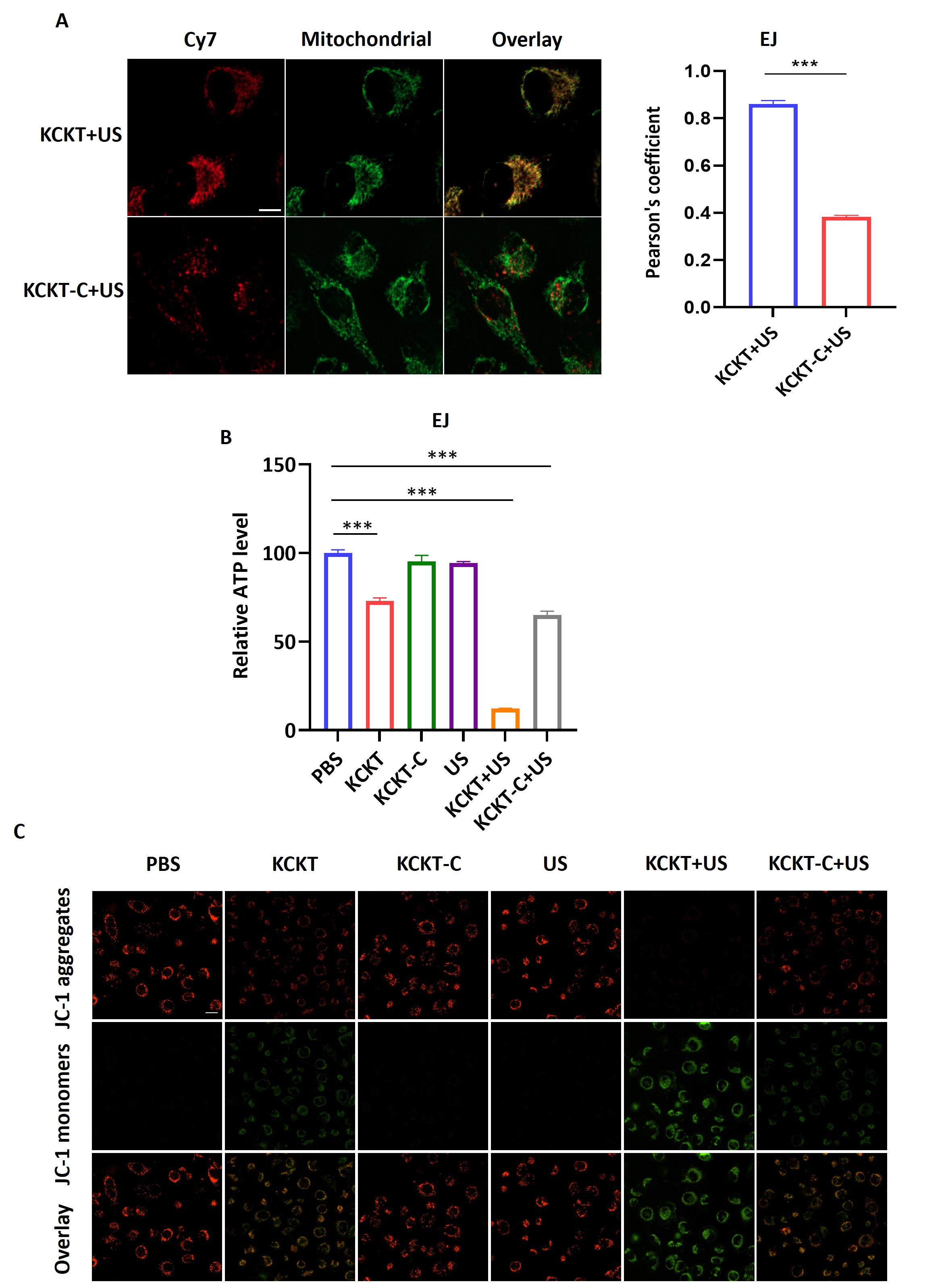


**Figure S8. The ability of KCKT to** **target and damage mitochondria were evaluated.** A) Confocal fluorescence images and co-localization coefficient analysis between nanoparticles and mitochondria in EJ cells treated with **KCKT+US** or **KCKT-C+US** (5 × 10^−6^ M). n = 3, Scale bar: 10 µm. B) Intracellular ATP levels and C) mitochondrial membrane potential in EJ cells after treated with PBS, **KCKT**, **KCKT-C**, US, **KCKT+US**, and **KCKT-C+US** (5 × 10^−6^ M). Scale bar: 20 µm. Statistical analyses were performed using Student’s t-test (A) and one-way ANOVA (B) with Bonferroni correction. Data presented as mean ± SEM. ***p < 0.001. US: 1.25 W cm^−2^, 1.0 MHz.


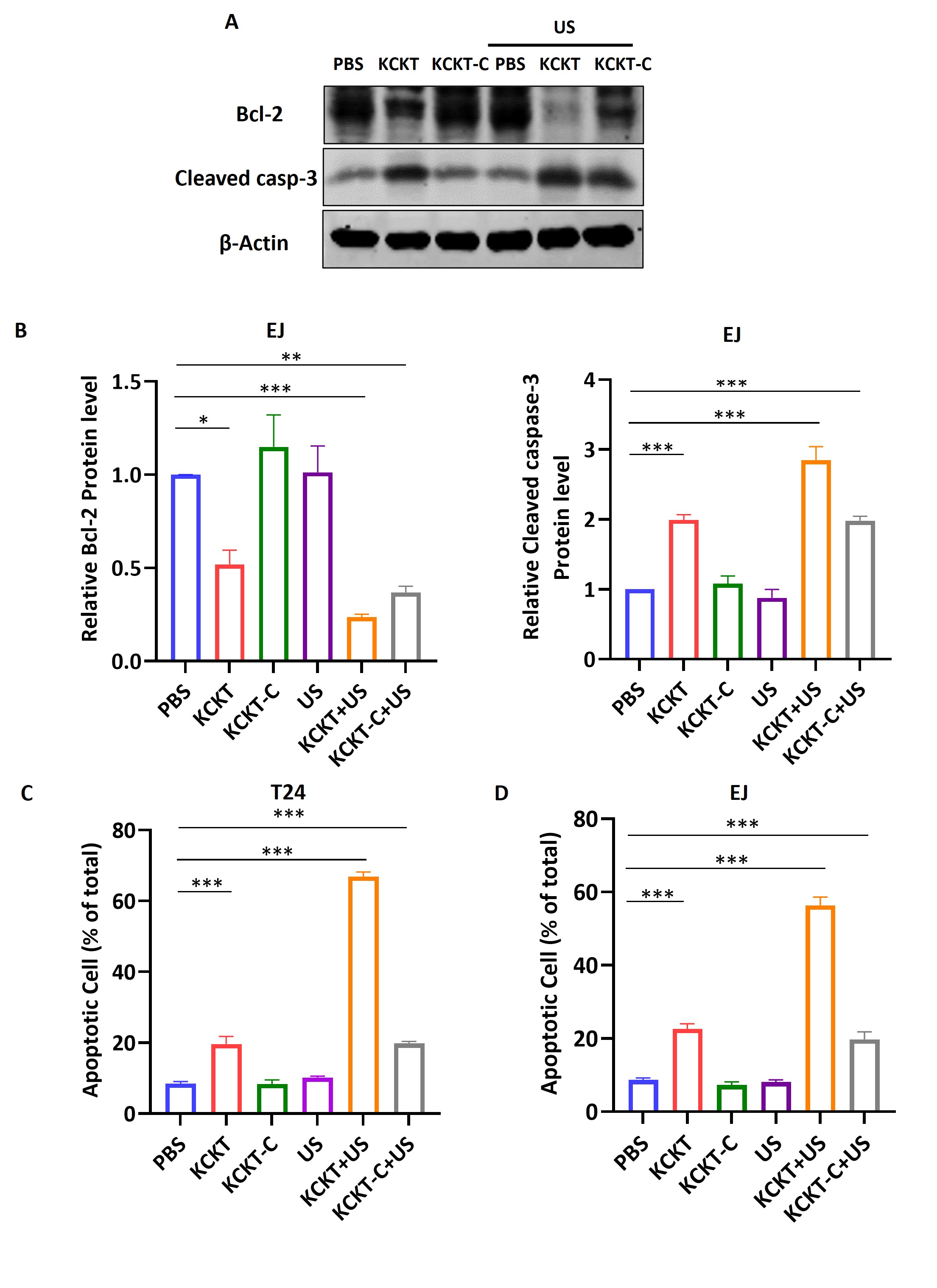


**Figure S9. The level of apoptosis in EJ and T24 cells after different treatment performances.** A) Western blot and B) quantitative analysis showing the expression levels of active cleaved Caspase-3 and the Bcl-2 in EJ cells. n = 3. C, D) The quantification of flow cytometry for T24 and EJ cells. n = 3. Statistical analyses were performed using one-way ANOVA with Bonferroni correction. Data presented as mean ± SEM. ***p < 0.001; **p < 0.01; *p < 0.05. US: 1.25 W cm^−2^, 1.0 MHz.


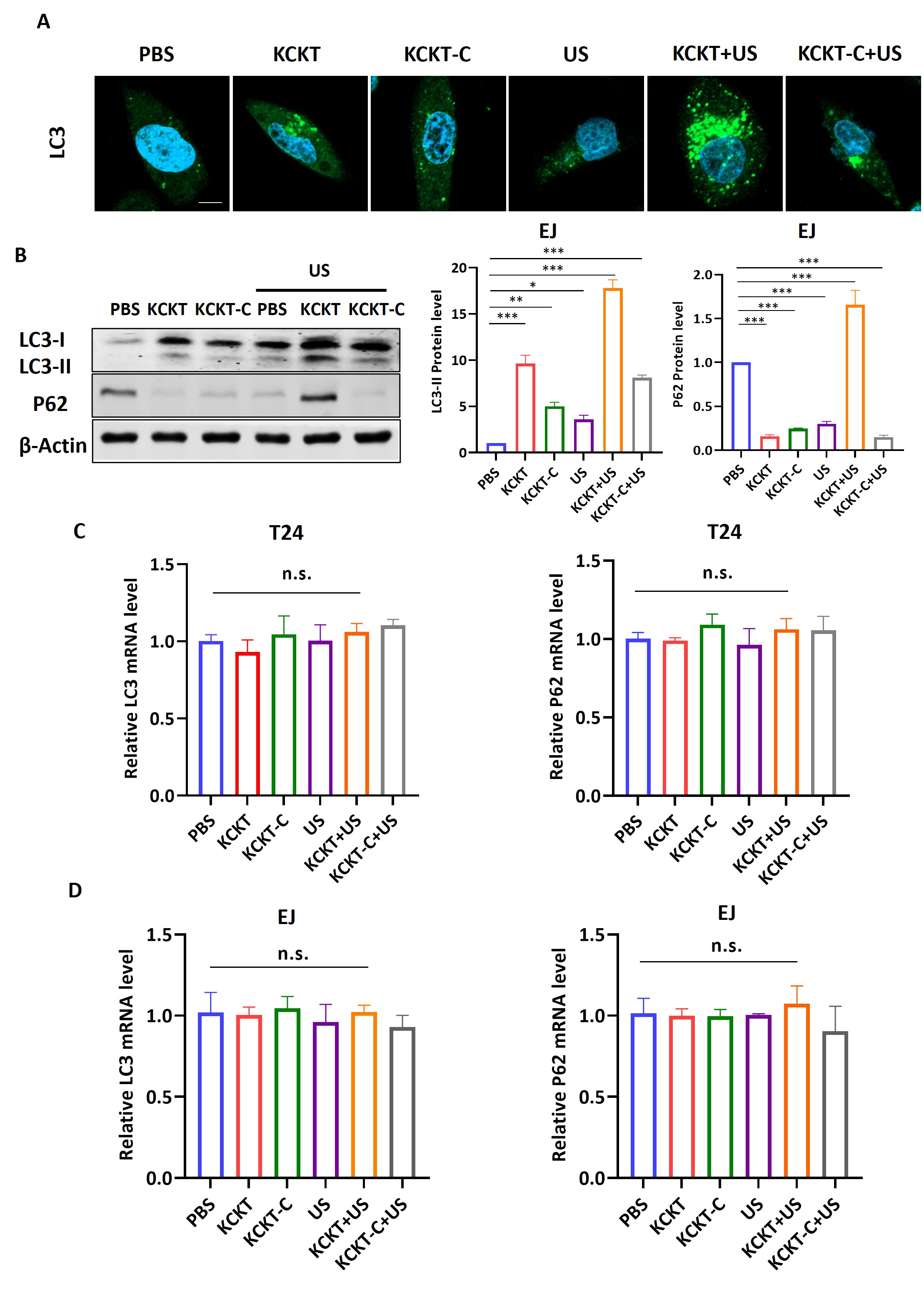


**Figure S10. The cellular autophagy level was examined after various treatments.** A) LC3 fluorescence intensity of EJ cells after treated with PBS, **KCKT**, **KCKT-C**, US, **KCKT+US**, and **KCKT-C+US** (5 × 10^−6^ M). Scale bar: 10 µm. B) The expression levels of LC3 and P62 in different treatments. n = 3. C, D) The analysis of the mRNA levels of LC3 and P62 in T24 and EJ cells. n = 4. Statistical analyses were performed using one-way ANOVA with Bonferroni correction. Data presented as mean ± SEM. ***p < 0.001; **p < 0.01; *p < 0.05. n.s. means no significance. US: 1.25 W cm^−2^, 1.0 MHz.


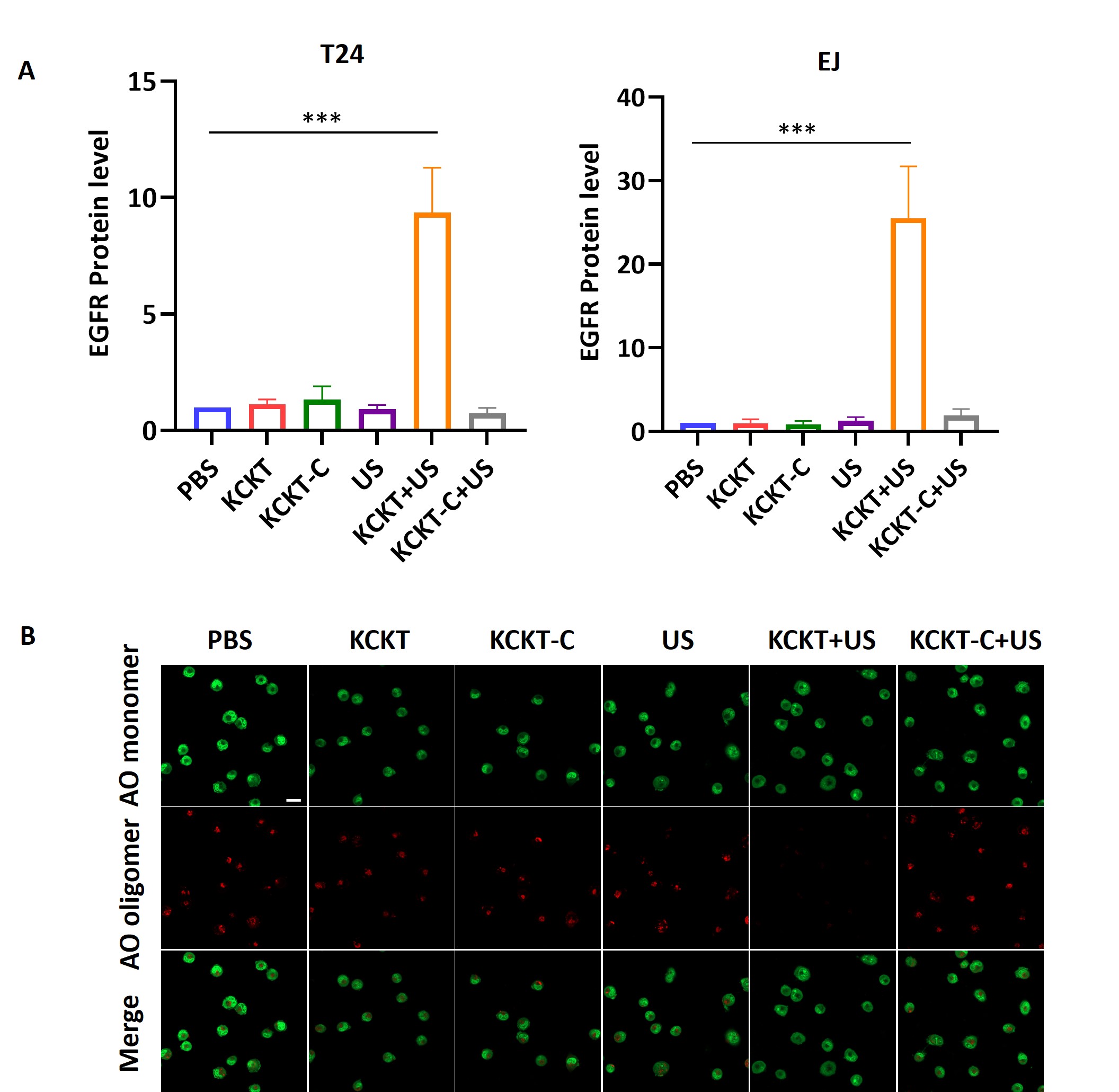


**Figure S11. Lysosomal functional detection against diverse treatments in T24 and EJ cells.** A) Quantitative analysis of EGFR protein in T24 and EJ cells treated with PBS, **KCKT**, **KCKT-C**, US, **KCKT+US** and **KCKT-C+US** (5 × 10^−6^ M). n = 3. B) Fluorescence images of EJ cells stained with AO after various treatments. Scale bar: 20 µm. Statistical analyses were performed using one-way ANOVA with Bonferroni correction. Data presented as mean ± SEM. ***p < 0.001. US: 1.25 W cm^−2^, 1.0 MHz.


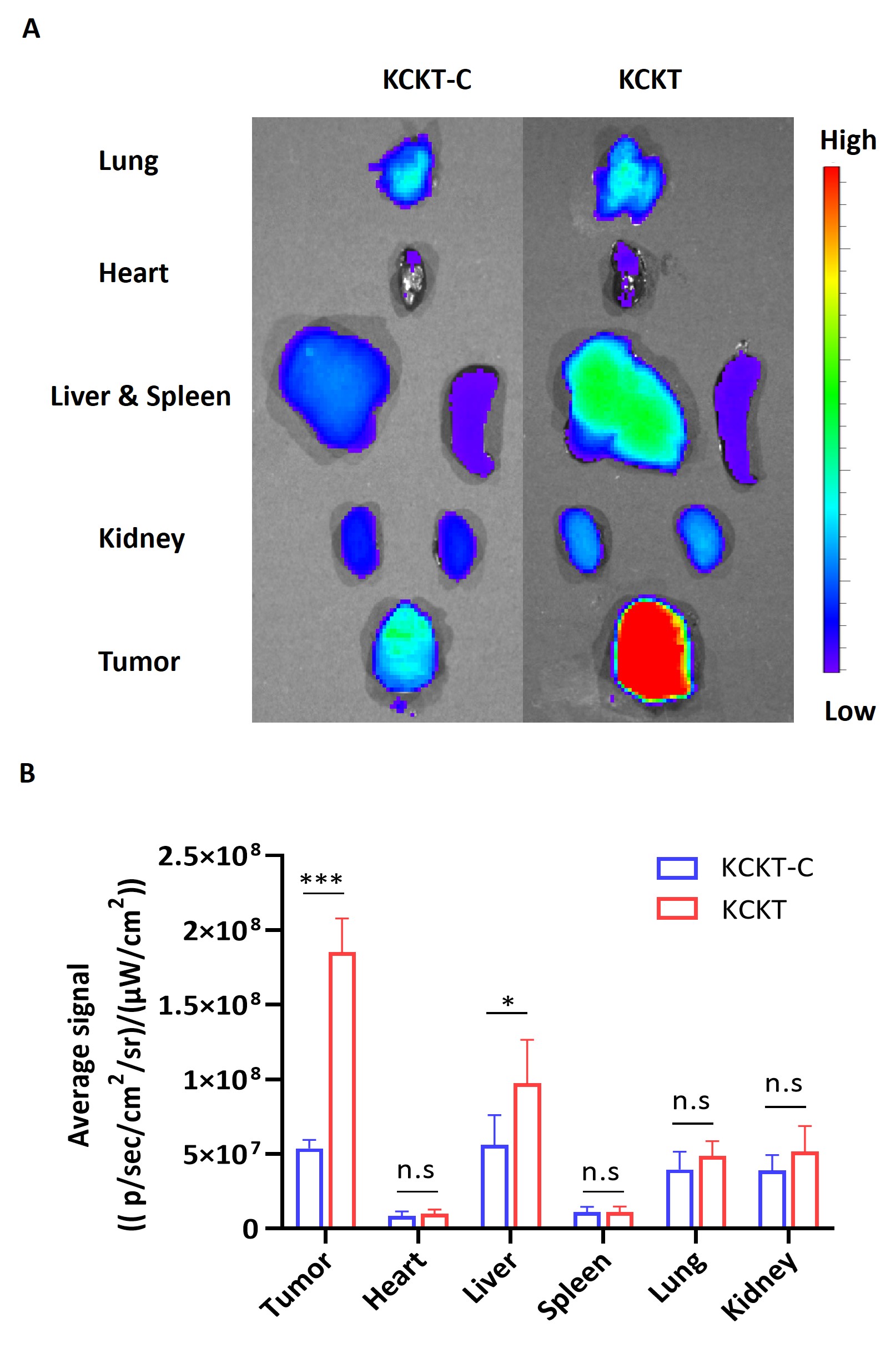


**Figure S12.** Fluorescence images and quantitative analysis of **KCKT** and **KCKT-C** in tumors and organs of mice. n = 6. Statistical analyses were performed using Student’s t-test. Data presented as mean ± SEM. ***p < 0.001; *p < 0.05., n.s. means no significance.


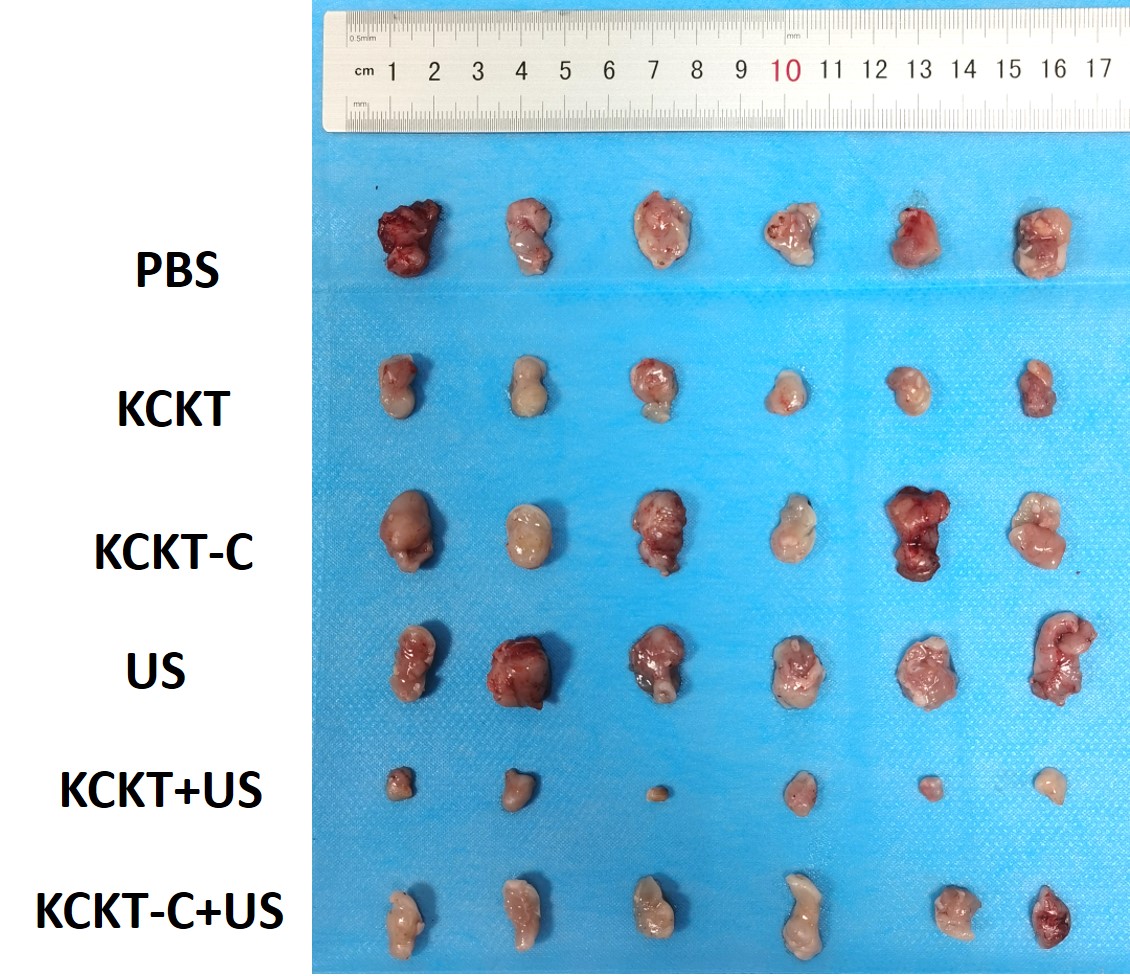


**Figure S13.** The representative photos of tumors were taken on day 28. n = 6. US: 1.25 W cm^−2^, 1.0 MHz.


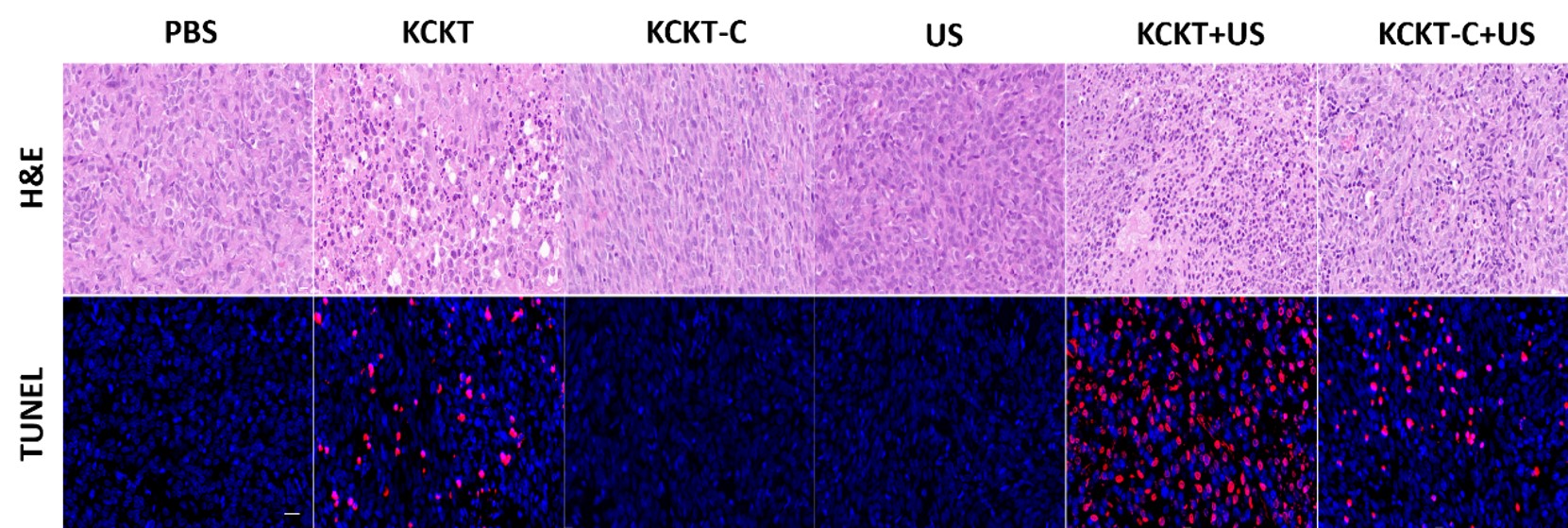


**Figure S14.** H&E and TUNEL staining of the tumor treated with PBS, **KCKT**, **KCKT-C**, US, **KCKT+US**, and **KCKT-C+US.** Scale bar: 10 µm. US: 1.25 W cm^−2^, 1.0 MHz.


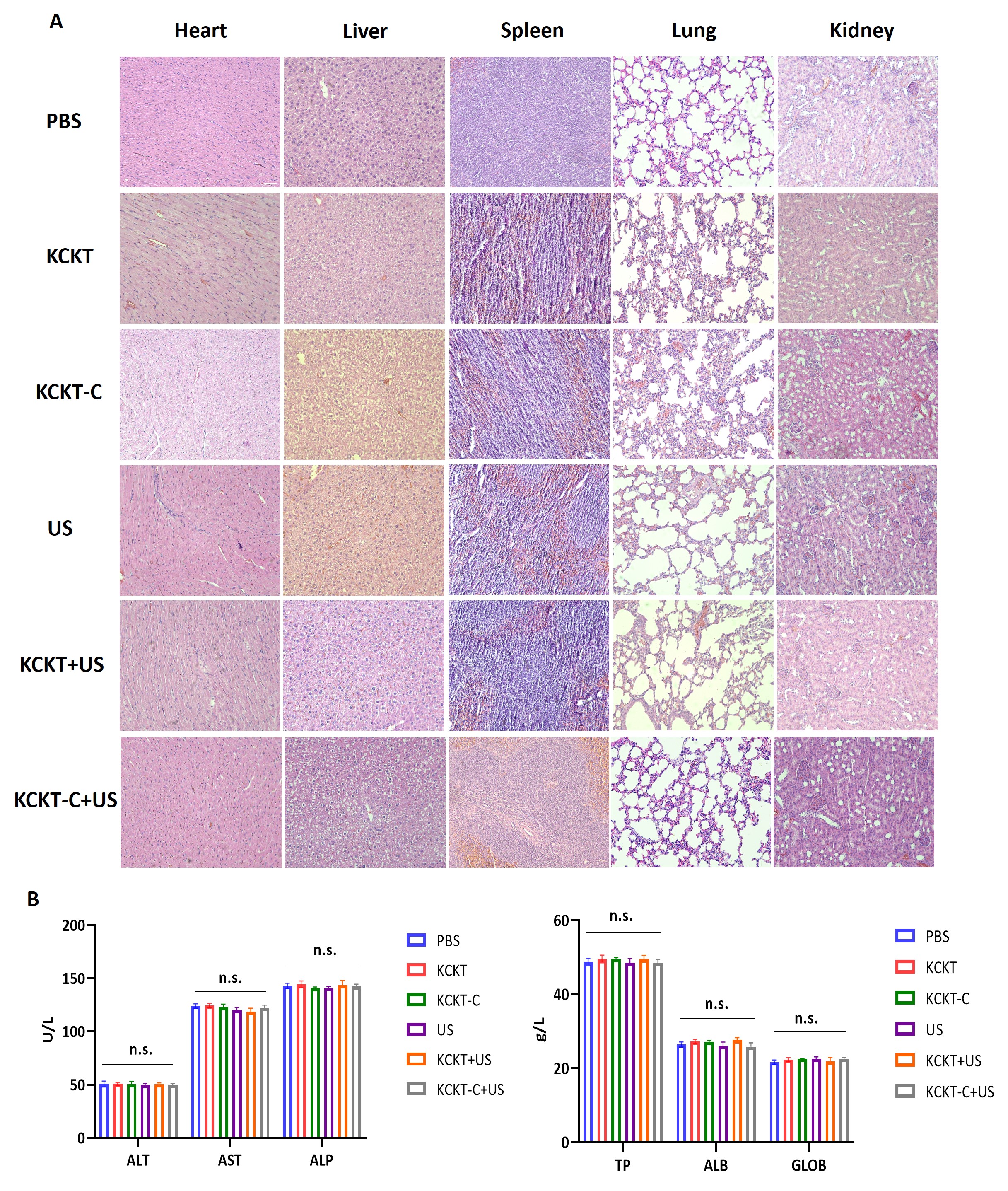


**Figure S15. The cytotoxicity of KCKT and KCKT-C in mice.** A) H&E staining of the heart, liver, spleen, lung, and kidney. Scale bar: 50 µm. B) Liver function test of mice treated with PBS, **KCKT**, **KCKT-C**, US, **KCKT+US**, and **KCKT-C+US**. ALT, Alanine aminotransferase; AST, Aspartate transaminase; ALP, Alkaline phosphatase; TP, Total protein; ALB, albumin; GLOB, globulin. n = 6. Statistical analyses were performed using one-way ANOVA with Bonferroni correction. Data presented as mean ± SEM. n.s. means no significance. US: 1.25 W cm^−2^, 1.0 MHz.
